# Supplementary figures and images for: Newcastle disease virus promotes spreading infection through vimentin-dependent tight junction injury mediated by MLC/p-MLC activation
Source: PLoS Pathog. 2025 Aug 29;21(8):e1013458. doi: 10.1371/journal.ppat.1013458 (PMC12410888; doi:10.1371/journal.ppat.1013458)

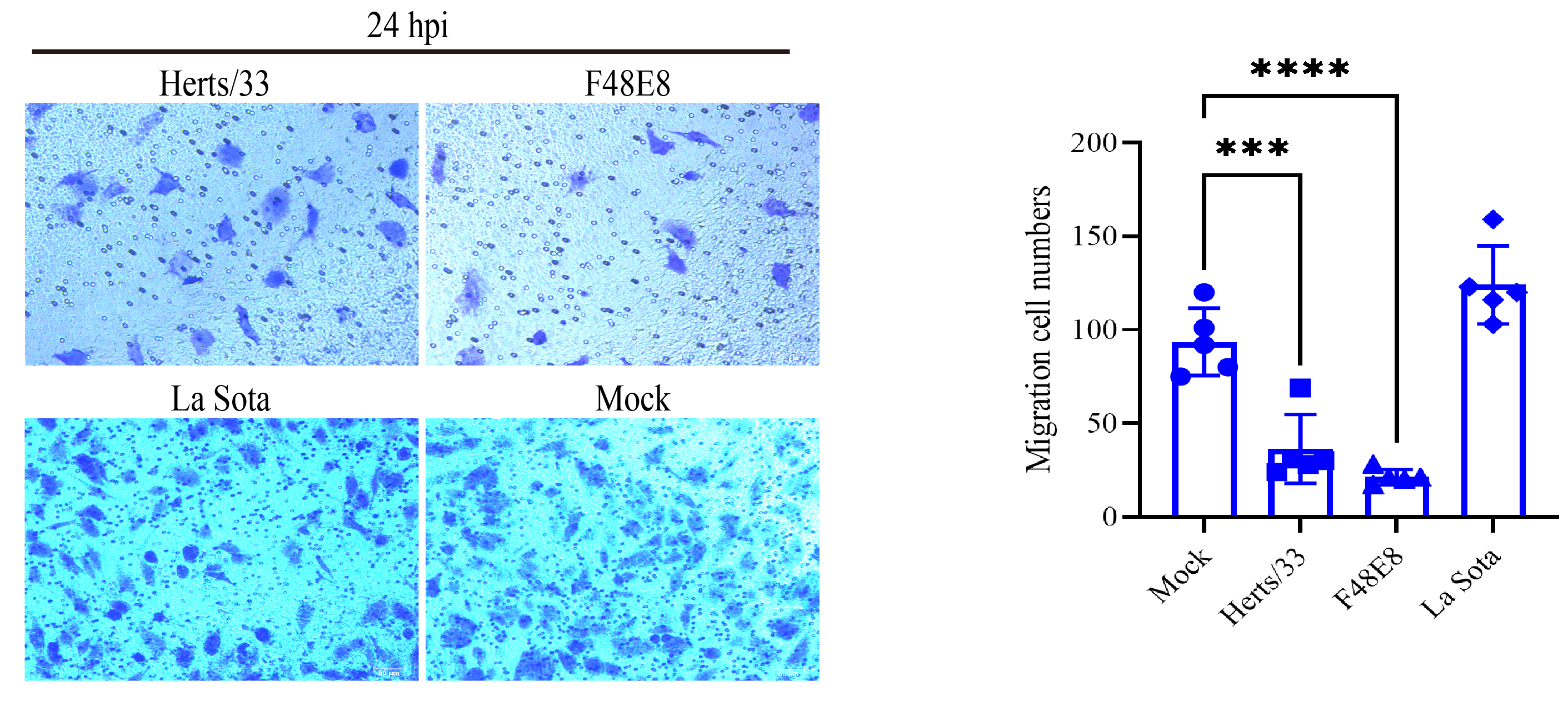

Supplement: S1 Fig — A transwell assay was performed on NDV- and mock-infected A549 cells (MOI = 0.1) at 24 hpi. The assay involved seeding 4 × 10⁴ cells in serum-free medium into the upper chamber, with the lower chamber supplemented with complete culture medium containing 20% fetal bovine serum to stimulate migration. Scale bar: 50 μm. *** P < 0.001; **** P < 0.0001. (TIF) [file ppat.1013458.s001.tif]

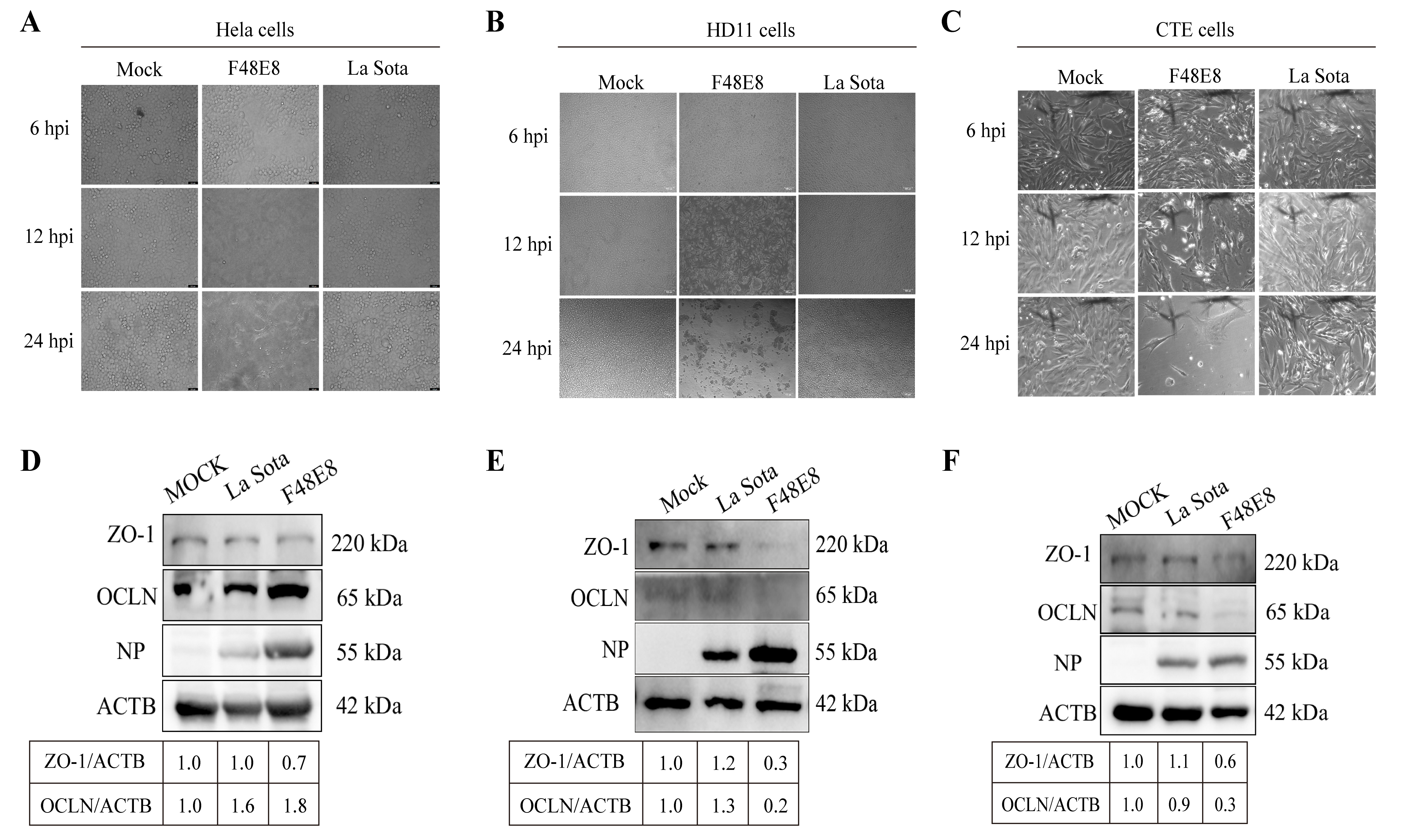

Supplement: S2 Fig — (A–C) The cellular morphology was monitored in NDV- and mock-infected HeLa, HD11, and CTE cells at an MOI of 1 from 6 to 24 hpi under light microscopy. Scale bar: 100 μm. (D–E) The expression levels of TJ-associated proteins OCLN and ZO-1 were assessed by western blotting at 24 hpi with NDV or mock (MOI = 1). The gray value of each protein was quantified by Image J and normalized to ACTB. The gray value of mock-infected group was considered as “1”, respectively. (TIF) [file ppat.1013458.s002.tif]

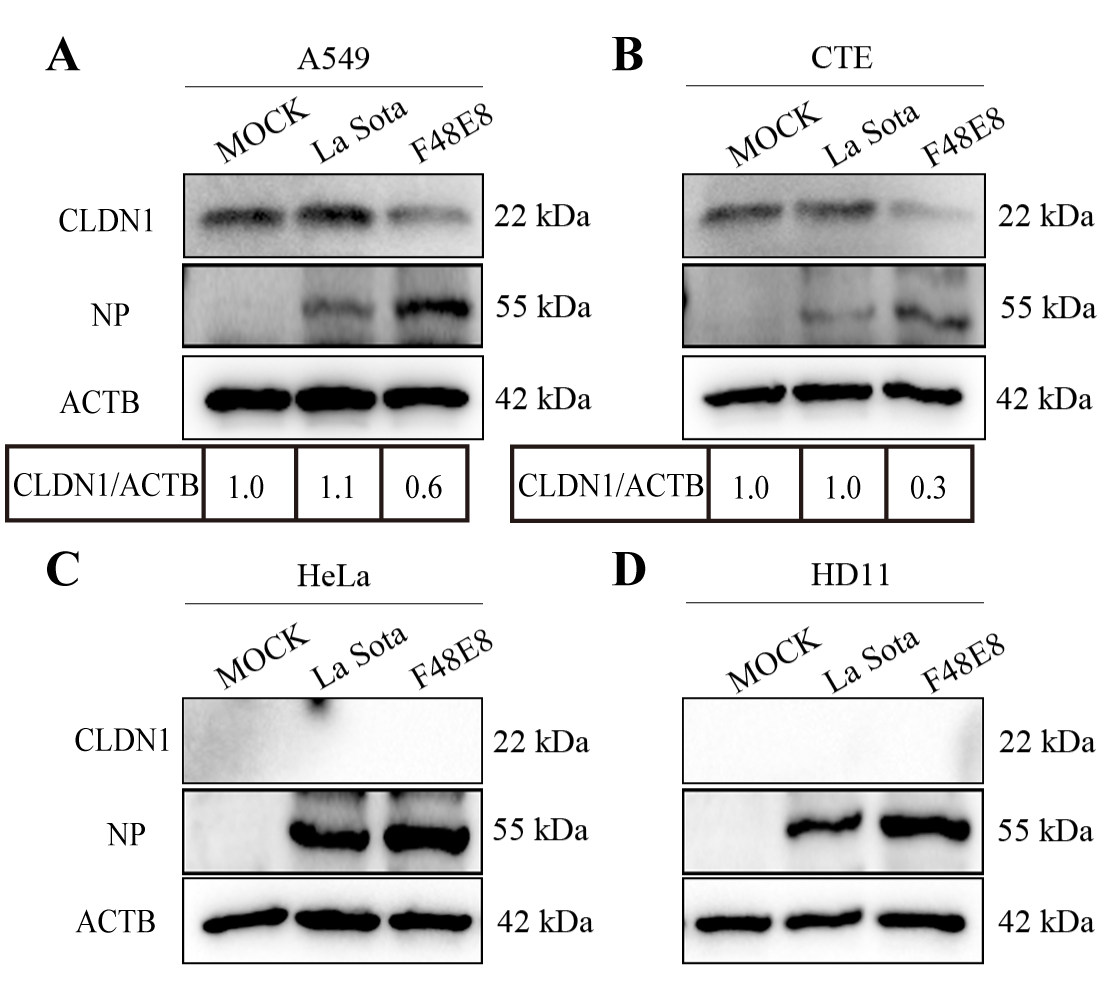

Supplement: S3 Fig — The expression levels of CLDN1 protein were assessed by western blotting at 24 hpi with NDV or mock (MOI = 1) in (A) A549, (B) CTE, (C) HeLa, and (D) HD11 cells. The gray value of each protein was quantified by Image J and normalized to ACTB. The gray value of mock-infected group was considered as “1”, respectively. (TIF) [file ppat.1013458.s003.tif]

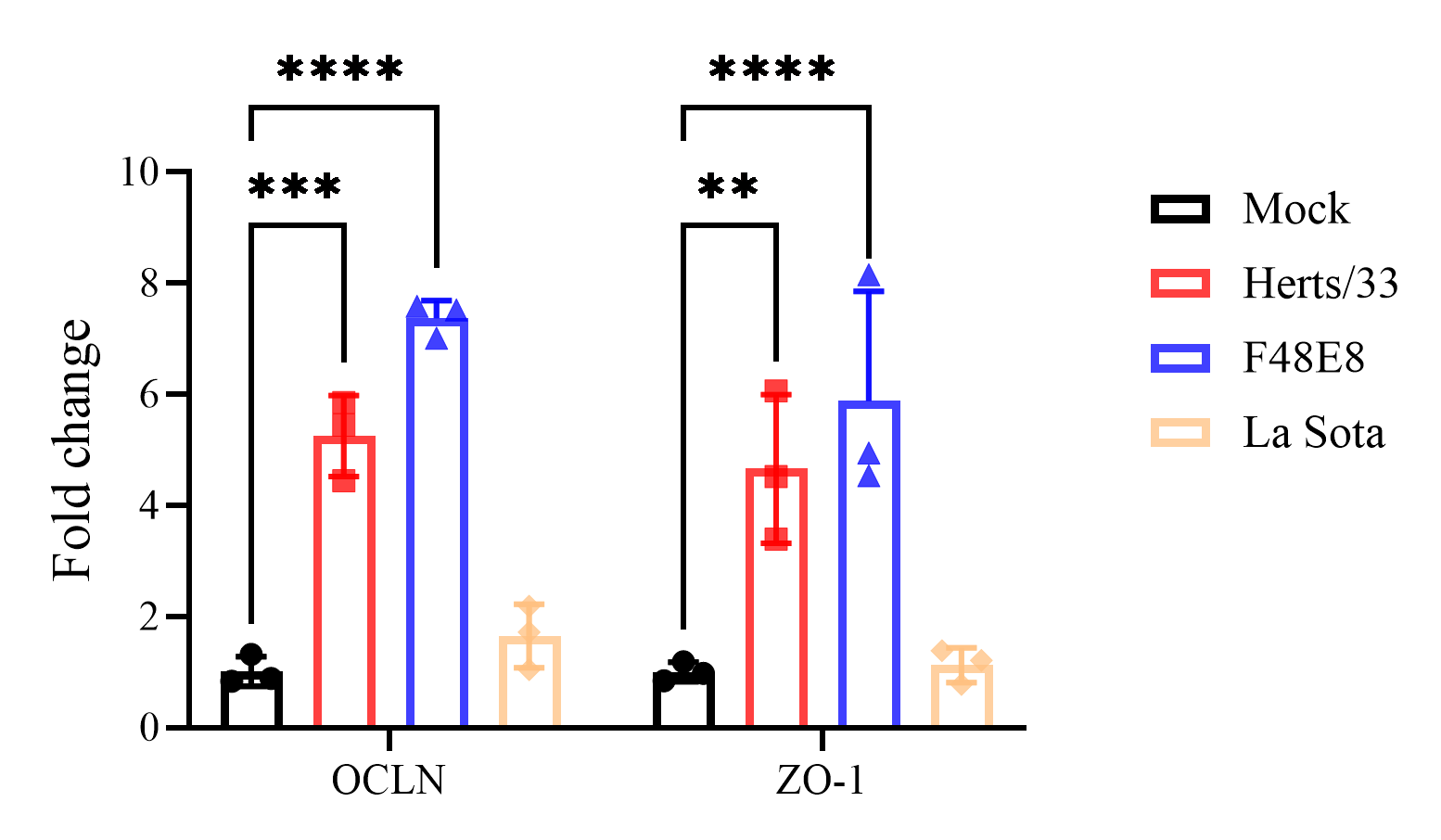

Supplement: S4 Fig — The mRNA levels of OCLN and ZO-1 genes were evaluated by qPCR following infection with NDV at 1 MOI for 24 h. NDV-infected groups were compared to the mock-infected group, and statistical analysis was carried out. ** P < 0.01; *** P < 0.001; **** P < 0.0001. (TIF) [file ppat.1013458.s004.tif]

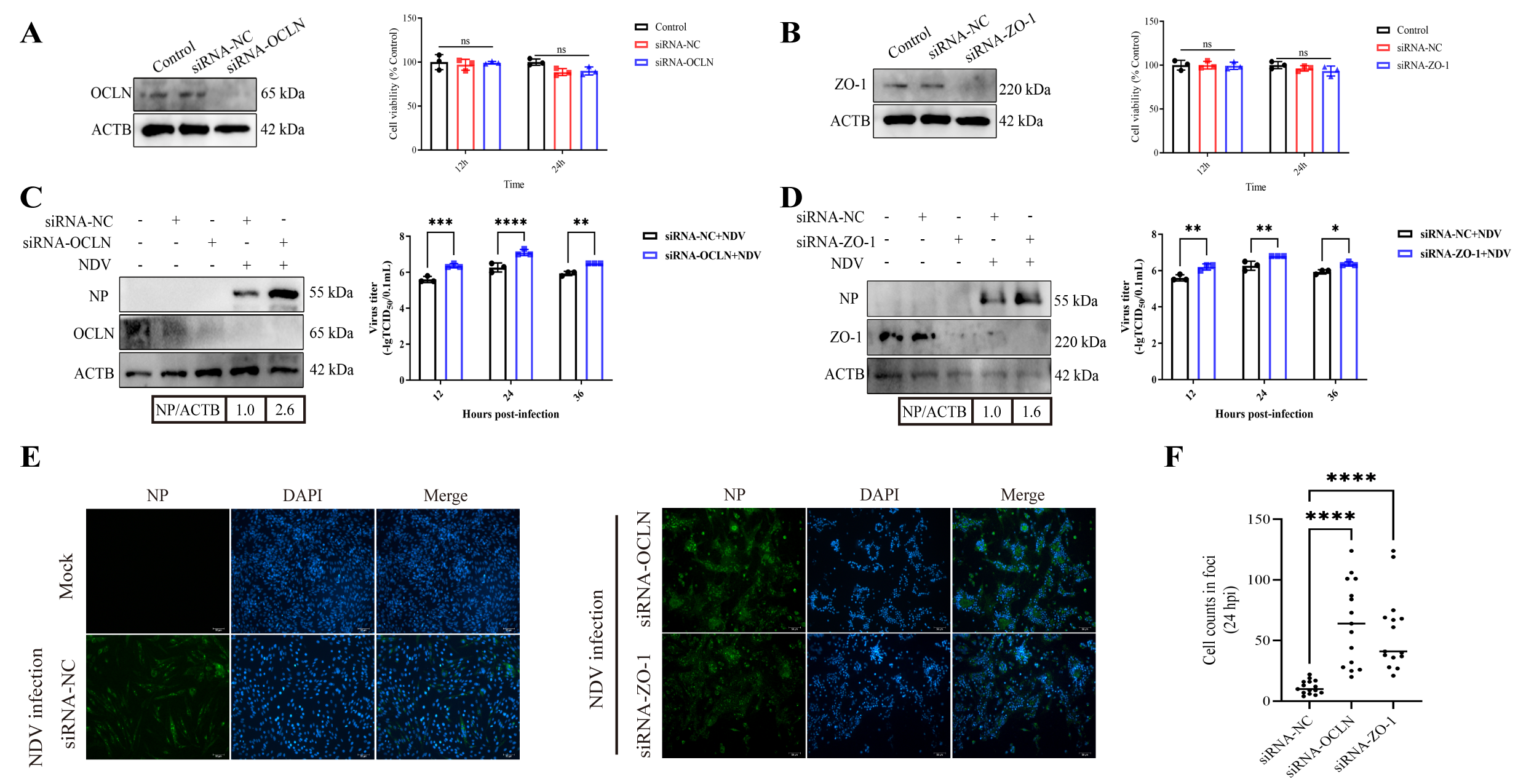

Supplement: S5 Fig — siRNA-OCLN and siRNA-ZO-1 (60 pmol) were employed to generate HD11/OCLN-KD and HD11/ZO-1-KD cells, respectively. siRNA-NC transfected cells were recognized as the control HD11/ctrl. (A) OCLN protein levels and cell viability were determined after siRNA transfection. The transfected groups were compared to the control group, and statistical analysis was performed. (B) ZO-1 protein levels and cell viability were determined after siRNA transfection. The transfected groups were compared to the control group, and statistical analysis was performed. (C) HD11/ctrl and HD11/OCLN-KD cells were infected with NDV at 1 MOI for 24 h. The viral replication in cells was evaluated by western blotting. The gray value of each protein was quantified by Image J and normalized to ACTB. The gray value of NDV-infected HD11/ctrl group was considered as “1”. Subsequently, these cells were infected with NDV at 0.1 MOI for 12, 24, and 36 h. The viral load in supernatants was detected by TCID50. (D) HD11/ctrl and HD11/ZO-1-KD cells were infected with NDV at 1 MOI for 24 h. The viral replication in cells was evaluated by western blotting. The gray value of each protein was quantified by Image J and normalized to ACTB. The gray value of NDV-infected HD11/ctrl group was considered as “1”. Subsequently, these cells were infected with NDV at 0.1 MOI for 12, 24, and 36 h. The viral load in supernatants was detected by TCID50. (E) HD11/ctrl, HD11/OCLN-KD and HD11/ZO-1-KD cells were infected with NDV at 0.00001 MOI for 24 h, and the foci in cells were evaluated by IFA. (F) Fifteen random lesions per treatment group were chosen, and the extent of viral dissemination was determined by counting the number of cells within each lesion. Scale bar: 50 μm. * P < 0.05; ** P < 0.01; *** P < 0.001; **** P < 0.0001; ns, no significant difference. (TIF) [file ppat.1013458.s005.tif]

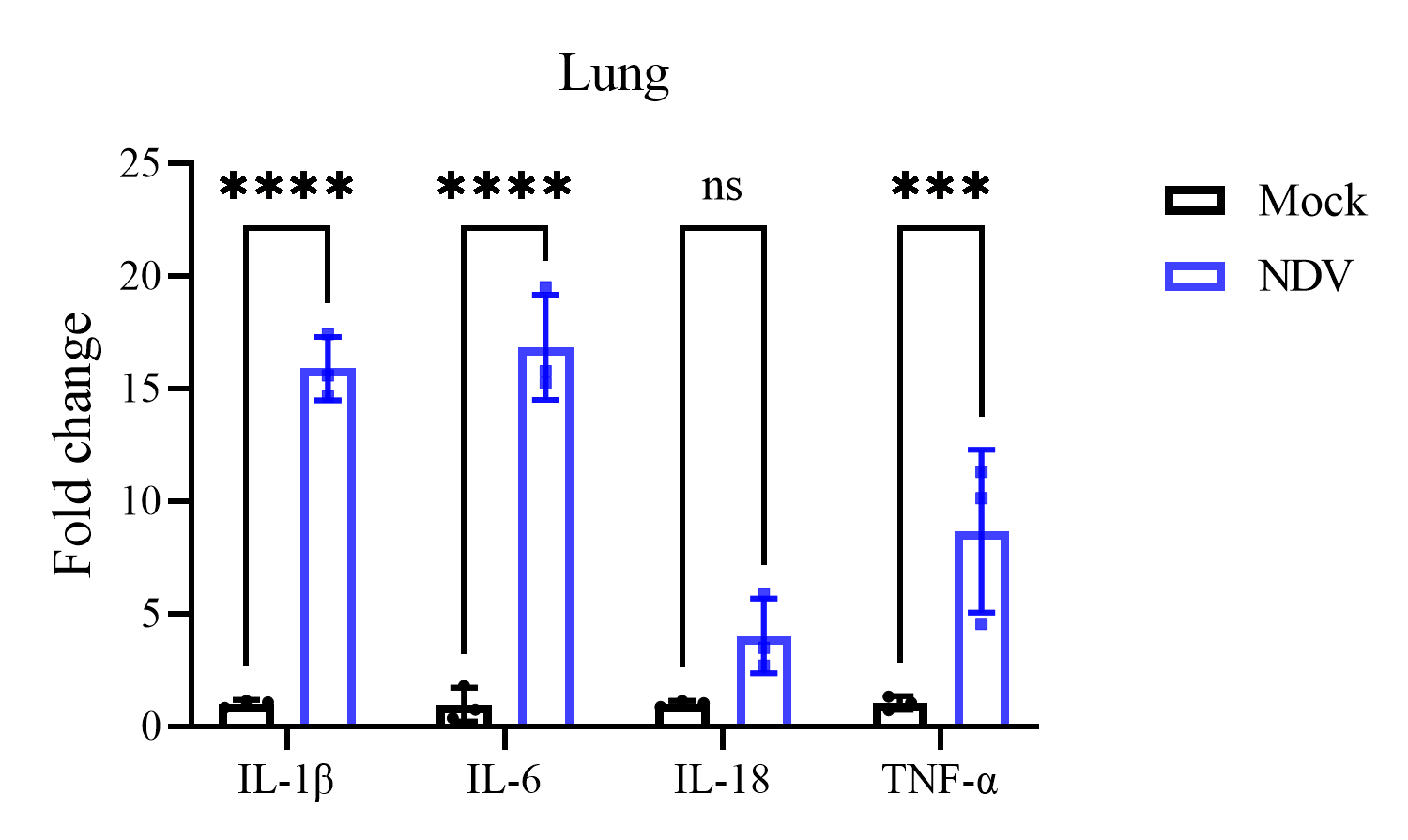

Supplement: S6 Fig — 4-week-old SPF chickens were infected with 105 EID50 NDV via the intranasal and intraocular route for 4 days. PBS group was considered as the control group. Lung tissue was then collected to measure the mRNA levels of inflammatory genes using qPCR. *** P < 0.001; **** P < 0.0001; ns, no significant difference. (TIF) [file ppat.1013458.s006.tif]

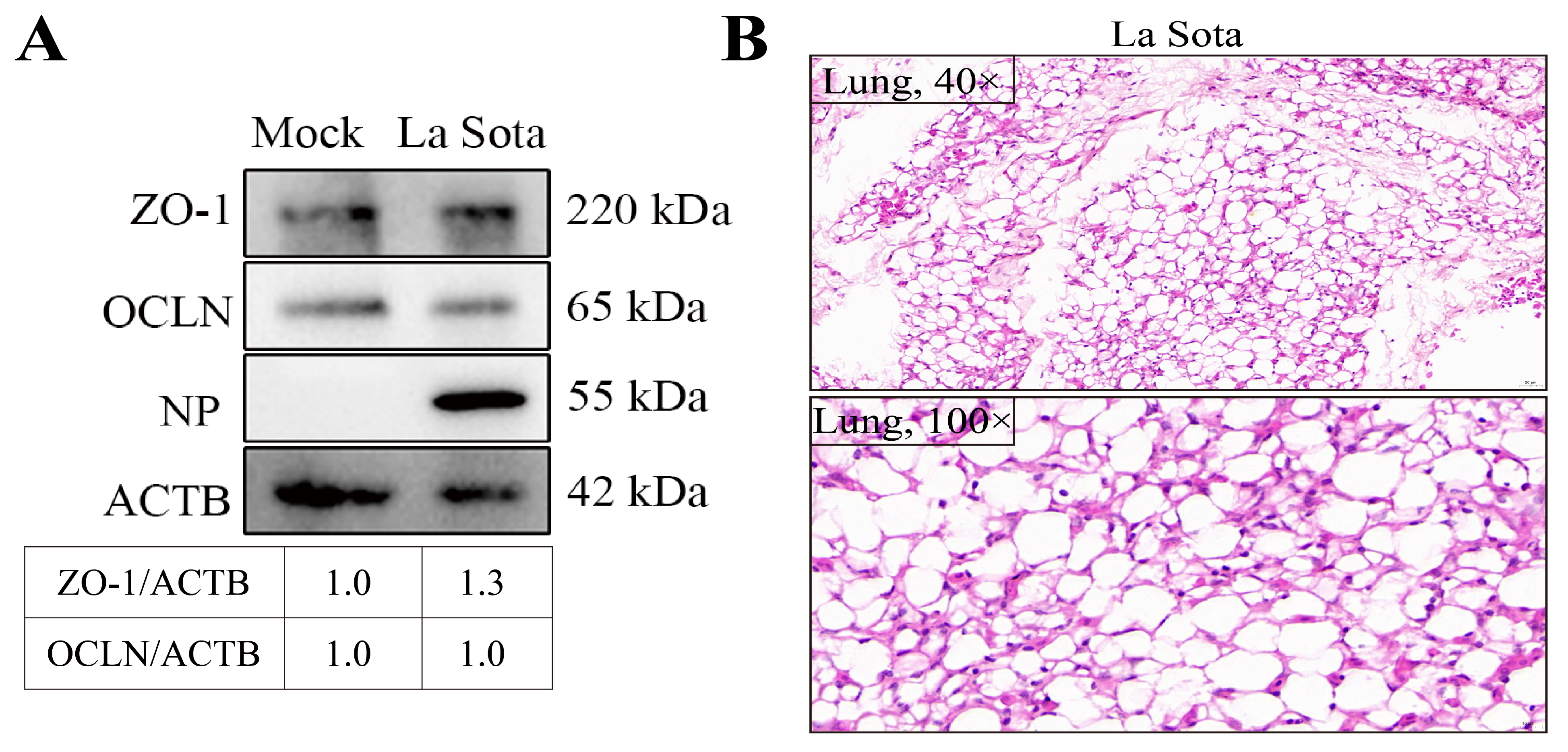

Supplement: S7 Fig — 4-week-old SPF chickens were infected with 105 EID50 La Sota via the intranasal and intraocular route for 4 days. PBS group was considered as the control group. (A) The lung tissue was harvested to detect OCLN and ZO-1 expression levels by western blotting. The gray value of each protein was quantified by Image J and normalized to ACTB. The gray value of mock-infected group was considered as “1”. (B) The histopathological alterations in chicken lung tissue post-La Sota infection were meticulously examined under 40× and 100 × magnification using H&E staining techniques. (TIF) [file ppat.1013458.s007.tif]

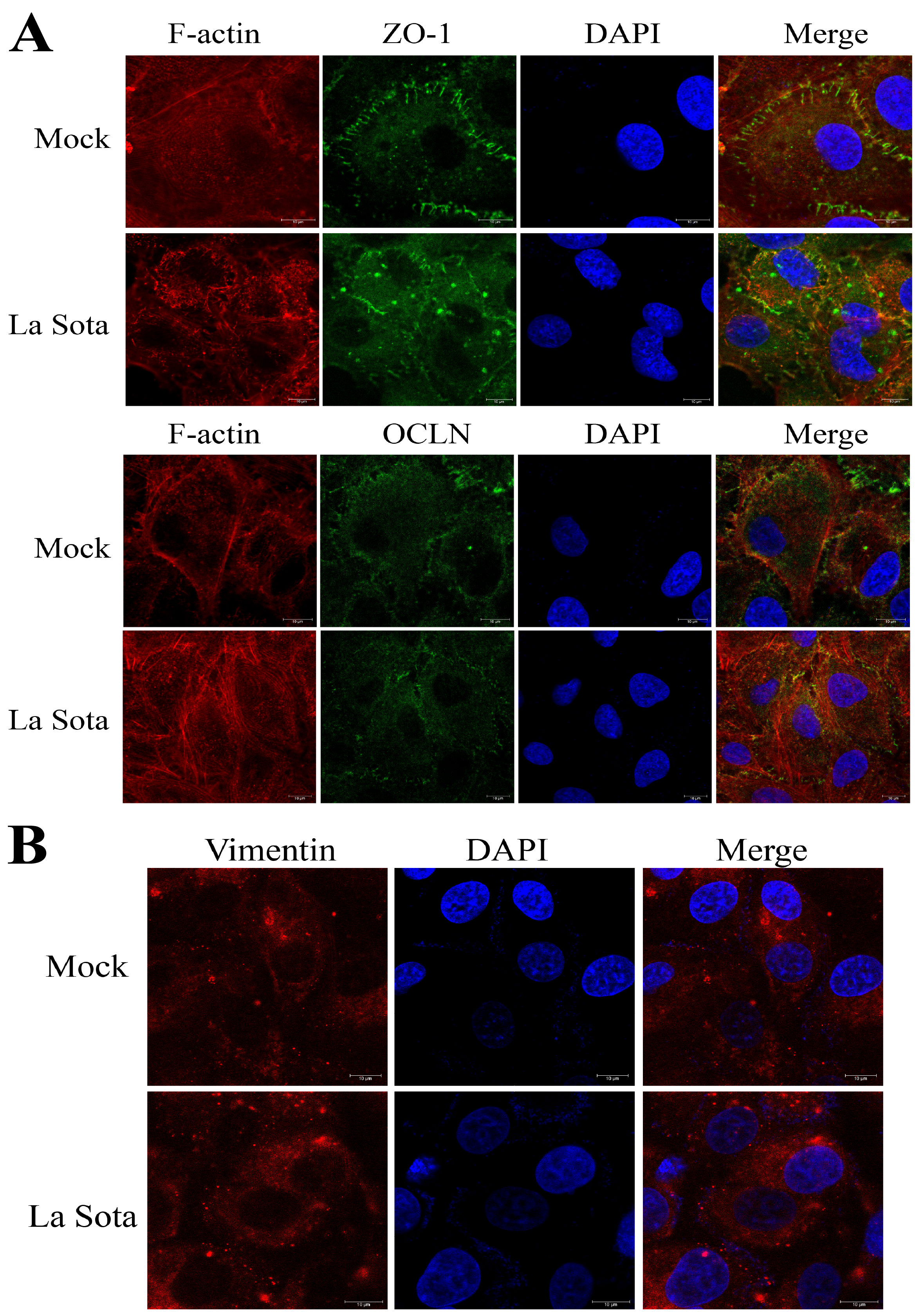

Supplement: S8 Fig — A549 cells were infected with La Sota at an MOI of 0.1 for 18 h. Mock group as a control for comparison. The localization of OCLN, ZO-1, F-actin (A), and vimentin (B) was observed by confocal microscopy. Scale bar: 10 μm. (TIF) [file ppat.1013458.s008.tif]

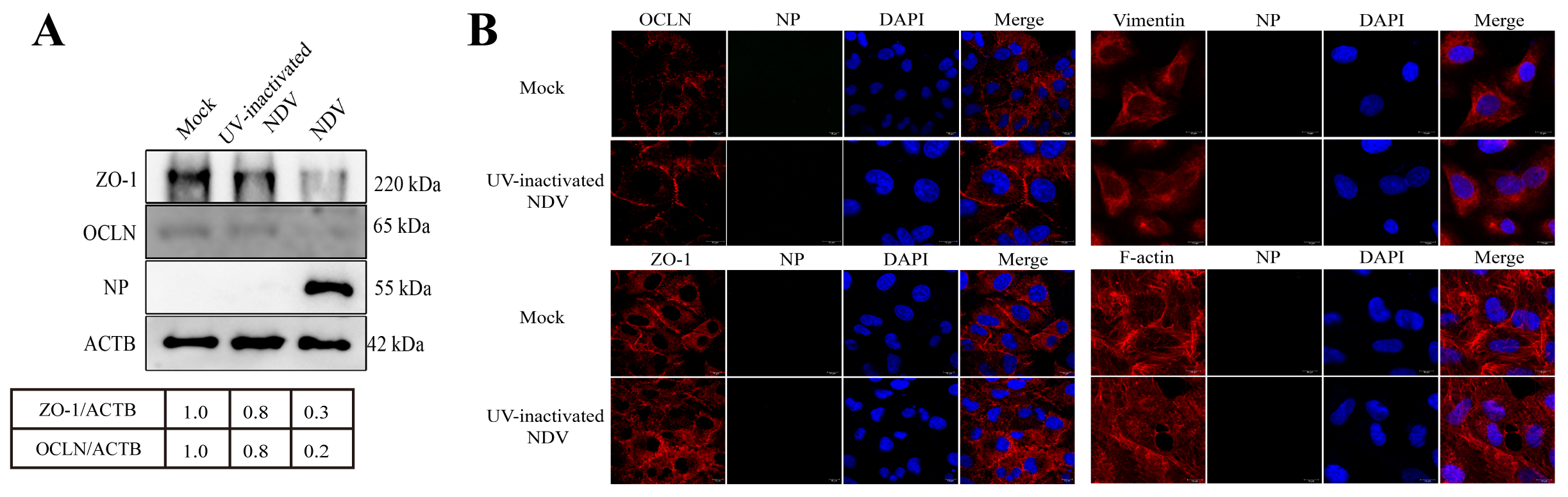

Supplement: S9 Fig — (A) OCLN and ZO-1 protein levels were assessed in A549 cells infected with either live NDV or UV-inactivated NDV (MOI = 1) by western blotting. The gray value of each protein was quantified by Image J and normalized to ACTB. The gray value of mock group was considered as “1”. (B) The localization of OCLN, ZO-1, vimentin, and F-actin was observed in A549 cells infected with either live NDV or UV-inactivated NDV at 0.1 MOI by confocal microscopy. Scale bar: 10 μm. (TIF) [file ppat.1013458.s009.tif]

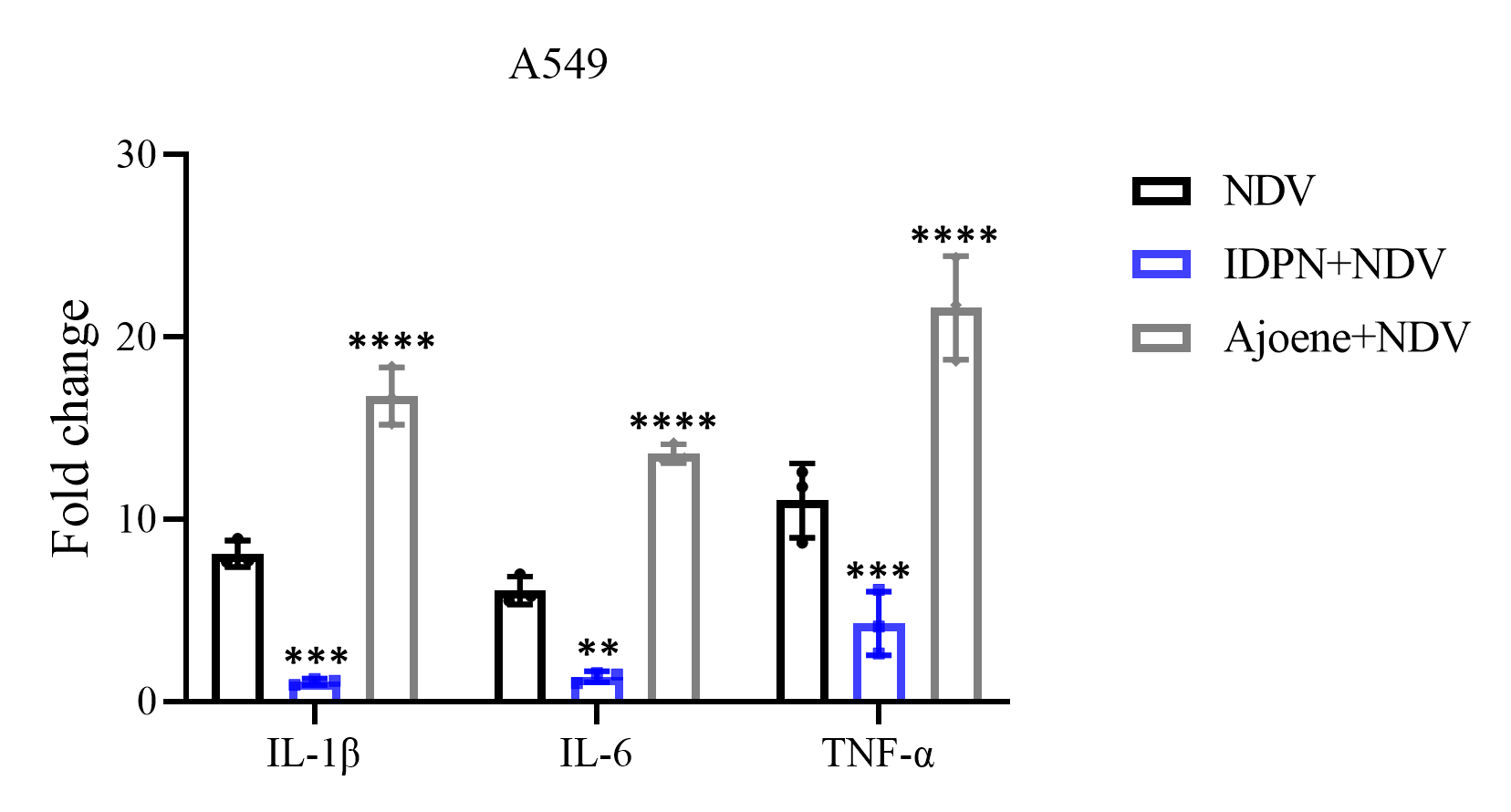

Supplement: S10 Fig — The mRNA levels of inflammatory genes (IL-1β, IL-6, and TNF-α) in NDV-infected A549 cells (MOI = 0.01) were detected by qPCR, comparing levels induced by IDPN or ajoene treatment. ** P < 0.01; *** P < 0.001; **** P < 0.0001. (TIF) [file ppat.1013458.s010.tif]

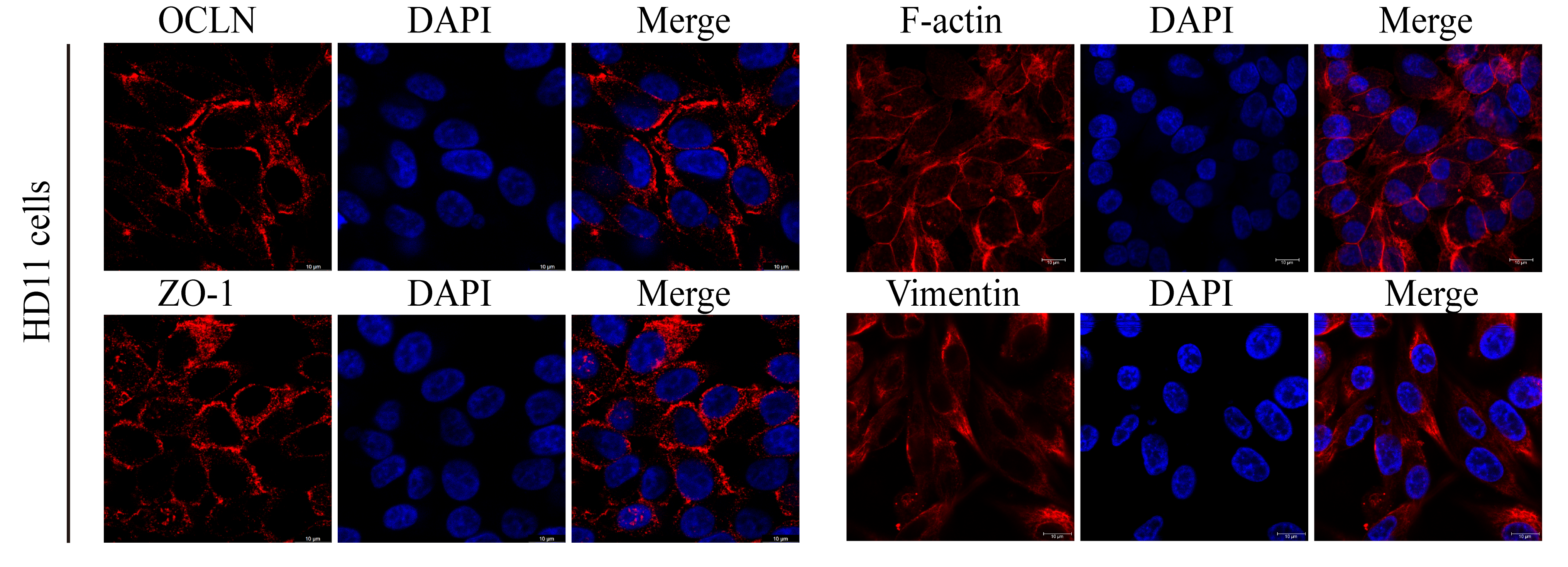

Supplement: S11 Fig — The structure of OCLN, ZO-1, F-actin, and vimentin was observed in normal HD11 cells. (TIF) [file ppat.1013458.s011.tif]

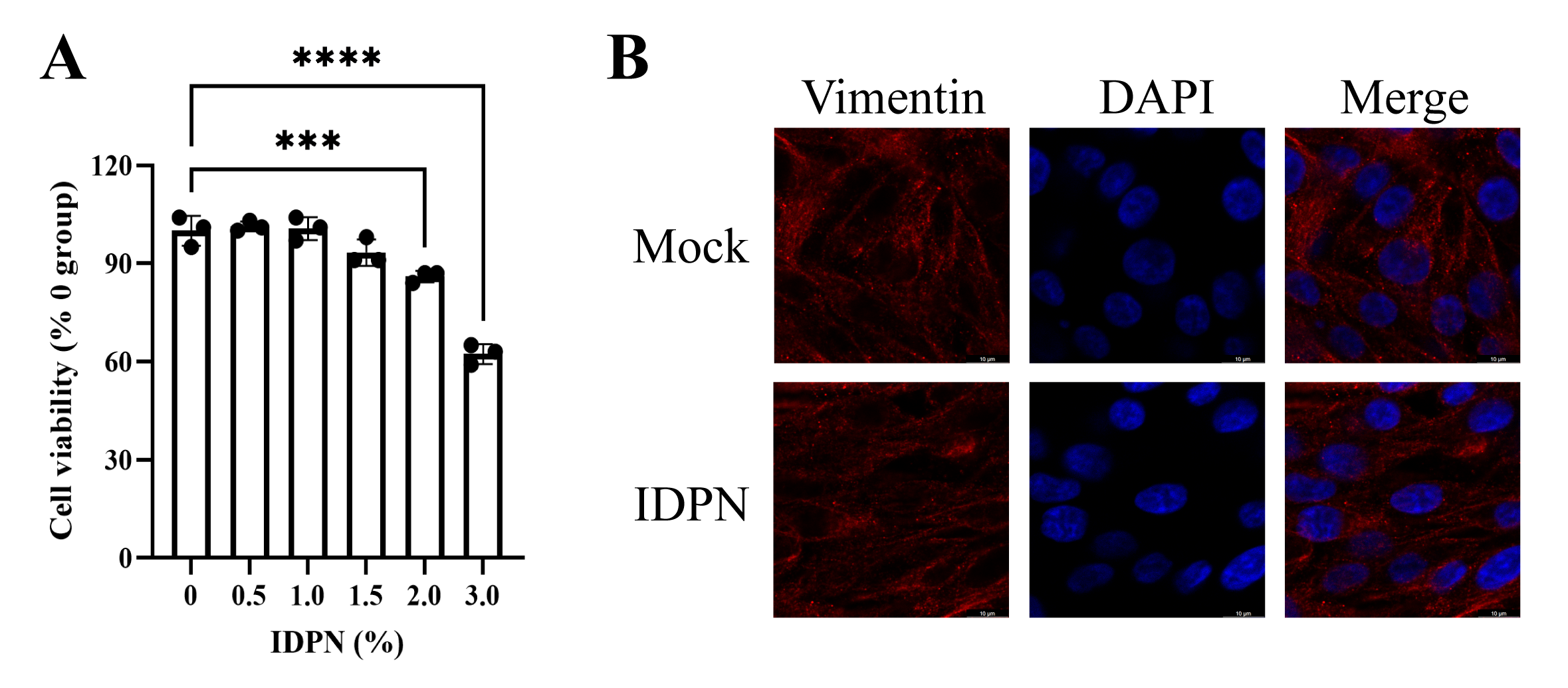

Supplement: S12 Fig — (A) Cytotoxicity of IDPN was assessed using the CCK8 assay. (B) Structural observation of vimentin in HD11 cells following IDPN treatment for 24 h. Scale bar: 10 μm. *** P < 0.001; **** P < 0.0001. (TIF) [file ppat.1013458.s012.tif]

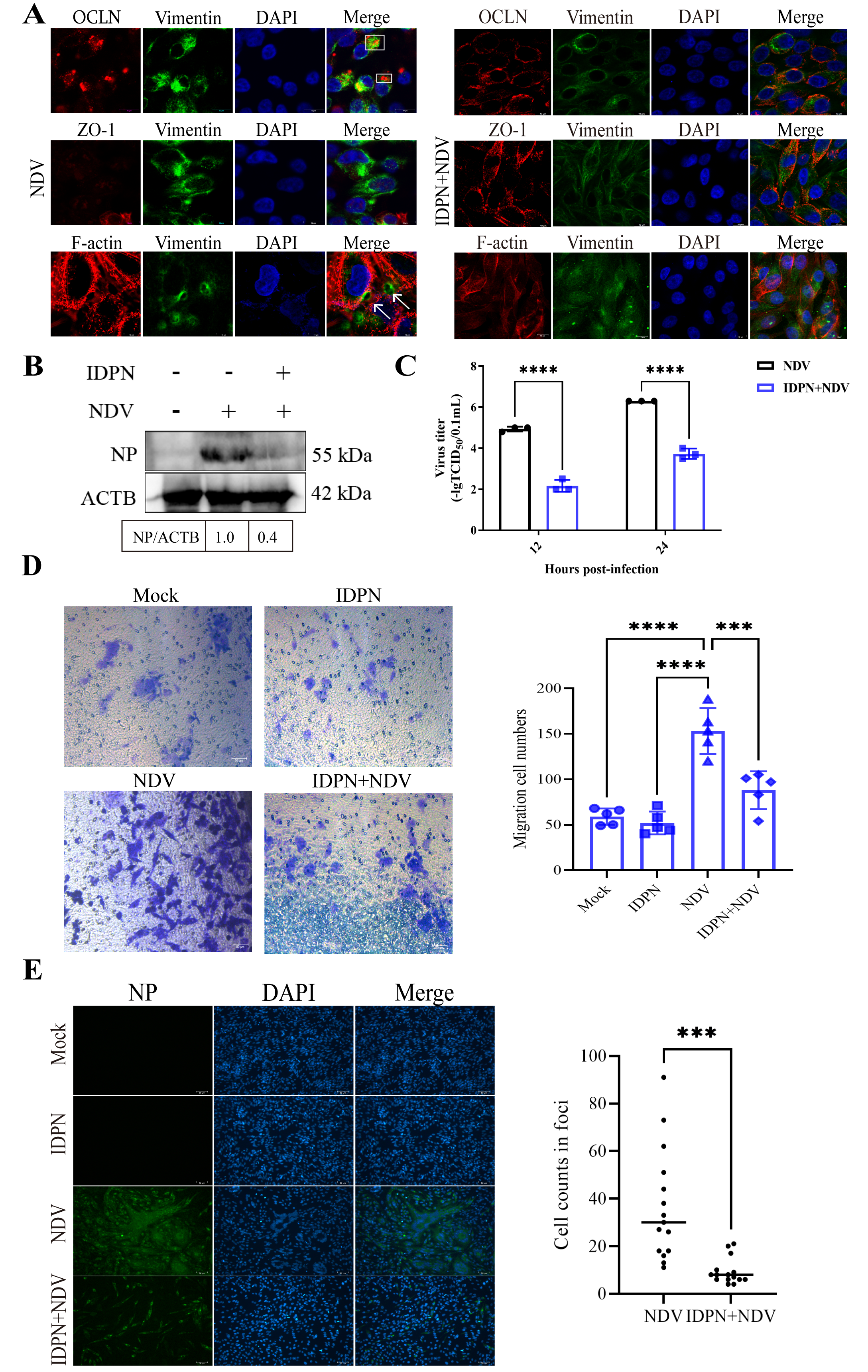

Supplement: S13 Fig — (A) The structure of OCLN, ZO-1, F-actin, and vimentin was observed in NDV-infected HD11 cells (MOI = 0.1) at 18 hpi following IDPN treatment (1.5%) by confocal microscopy. The white arrows represent the vimentin and F-actin cages, whereas the dashed box highlights the OCLN aggregates in vimentin cages. Scale bar: 10 μm. (B) The replication levels of NDV (MOI = 0.1) were quantified by western blotting at 24 hpi, comparing NP levels in the presence and absence of IDPN (1.5%). The gray value of each protein was quantified by Image J and normalized to ACTB. The gray value of NDV-infected group was considered as “1”. (C) The replication levels of NDV (MOI = 0.1) were quantified using the TCID50 assay at 12 and 24 hpi, comparing the viral titers in the presence and absence of IDPN (1.5%). (D) The cell migration by NDV (MOI = 0.1) were assessed at 6 hpi using the transwell assay, comparing the amount of migrated HD11 cells in the presence and absence of IDPN (1.5%). Scale bar: 50 μm. (E) The cell foci were measured in NDV-infected (MOI = 0.00001) HD11 cells at 36 hpi by IFA, comparing the number of cells within foci in the presence and absence of IDPN (1.5%). Scale bar: 50 μm. *** P < 0.001; **** P < 0.0001. (TIF) [file ppat.1013458.s013.tif]

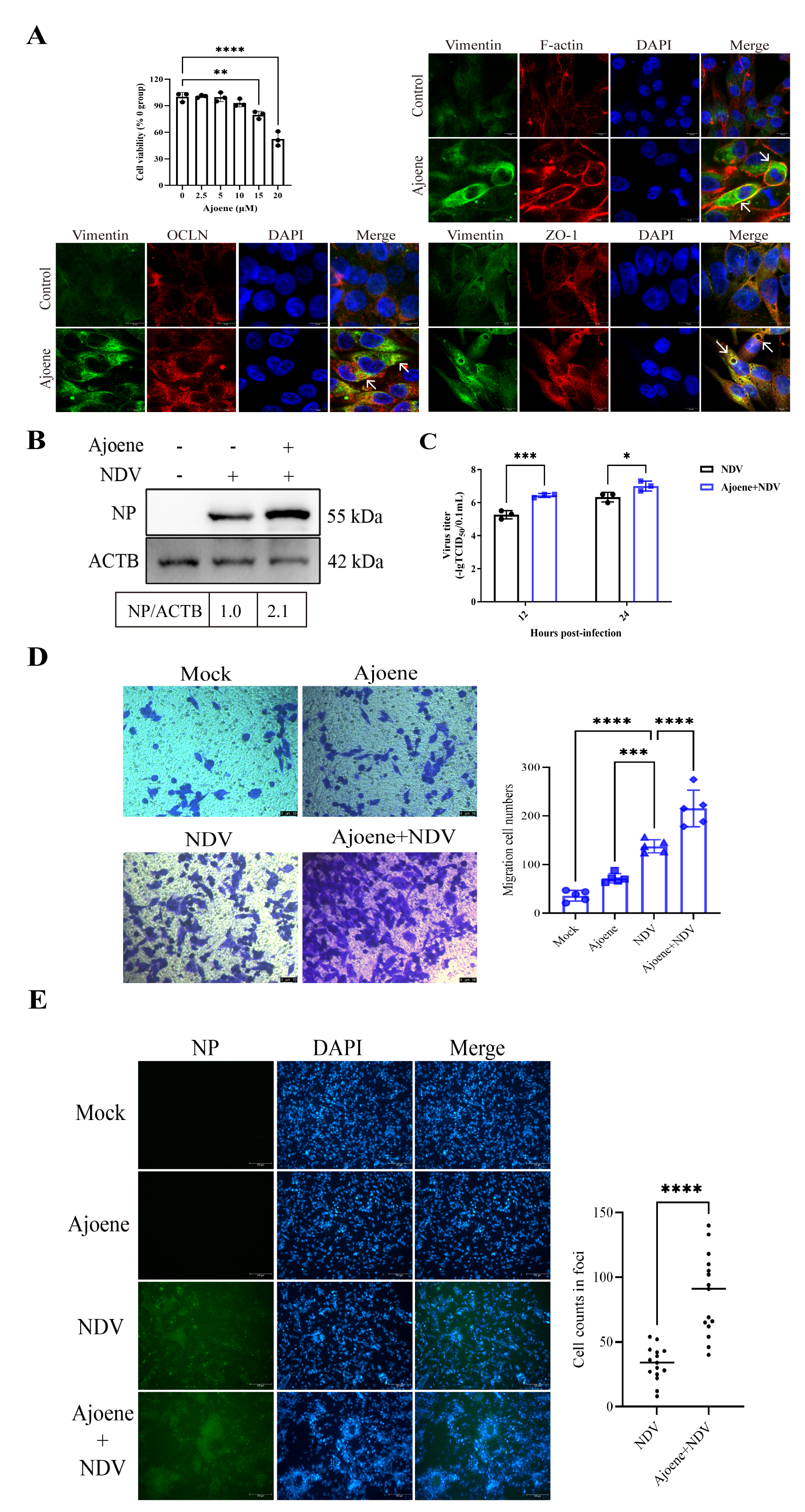

Supplement: S14 Fig — (A) Cytotoxicity of ajoene was assessed using the CCK8 assay. Structural examination of vimentin, F-actin, OCLN, and ZO-1 in HD11 cells treated with ajoene (10 μM) was conducted. The white arrow indicates the rearranged structure. Scale bar: 10 μm. (B) The NP protein levels were examined in NDV-infected (MOI = 1) HD11 cells treated with or without ajoene (10 μM) by western blotting. The gray value of each protein was quantified by Image J and normalized to ACTB. The gray value of NDV-infected group was considered as “1”. (C) The replication levels of NDV (MOI = 0.1) were quantified using the TCID50 assay in HD11 cells, comparing the viral titers in the presence and absence of ajoene (10 μM). (D) The cell migration by NDV (MOI = 0.1) were assessed at 6 hpi using the transwell assay in HD11 cells, comparing the amounts of migrated cells in the presence and absence of ajoene (10 μM). Scale bar: 50 μm. (E) The cell foci were measured in NDV-infected HD11 cells (MOI = 0.00001) by IFA, comparing the number of cells within foci in the presence and absence of ajoene (10 μM). Scale bar: 50 μm. * P < 0.05; ** P < 0.01; *** P < 0.001; **** P < 0.0001. (TIF) [file ppat.1013458.s014.tif]

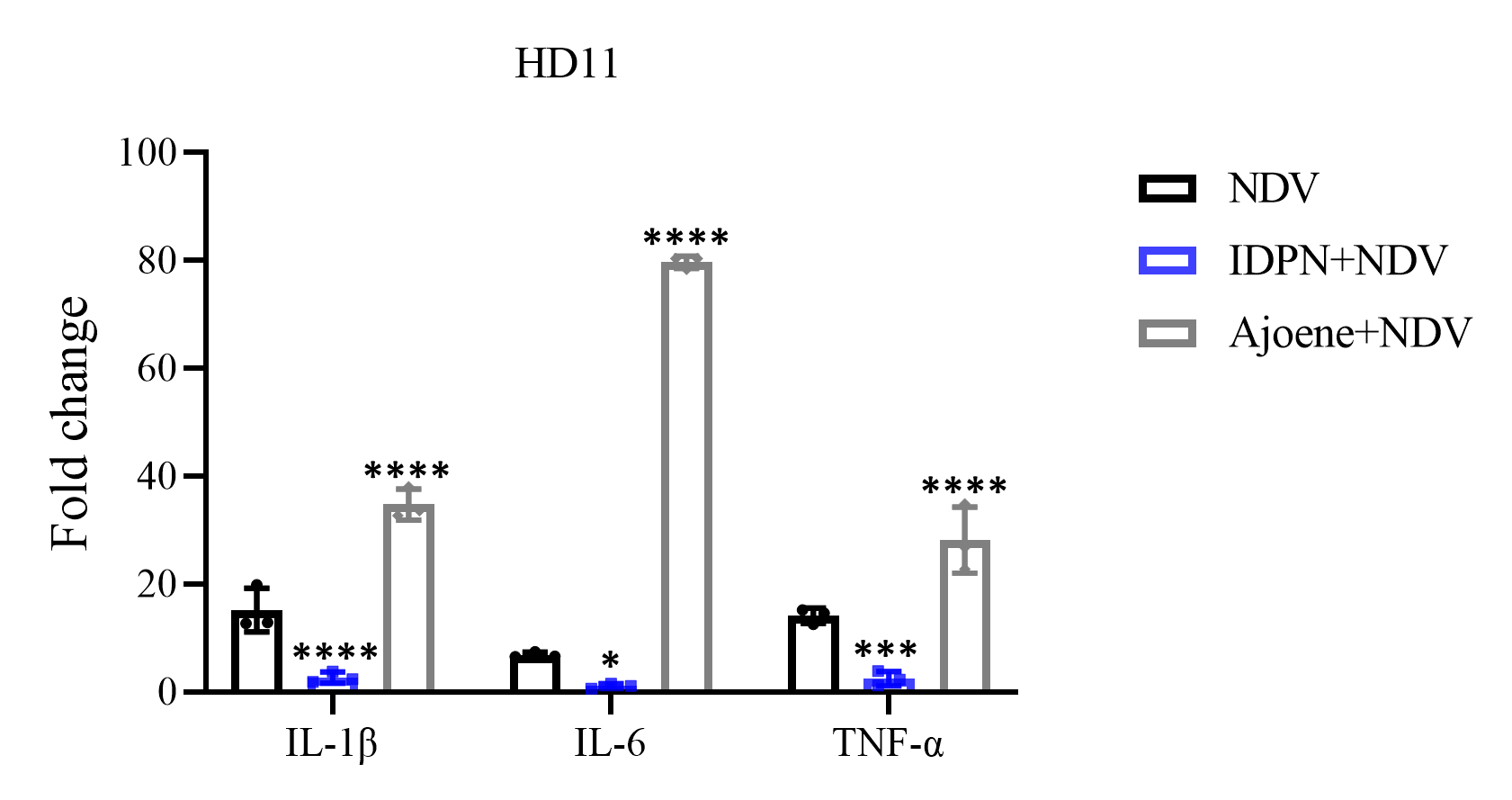

Supplement: S15 Fig — The mRNA levels of inflammatory genes (IL-1β, IL-6, and TNF-α) in NDV-infected HD11 cells (MOI = 0.01) were detected by qPCR, comparing levels induced by IDPN (1.5%) or ajoene (10 μM) treatment. * P < 0.05; *** P < 0.001; **** P < 0.0001. (TIF) [file ppat.1013458.s015.tif]
