# Supplementary material for: Newcastle disease virus promotes spreading infection through vimentin-dependent tight junction injury mediated by MLC/p-MLC activation
Source: PLoS Pathog. 2025 Aug 29;21(8):e1013458. doi: 10.1371/journal.ppat.1013458 (PMC12410888; doi:10.1371/journal.ppat.1013458)
Supplement: S1 Table — (DOCX) [file ppat.1013458.s016.docx]

**S1 Table.** Characteristics of NDV strains used in this study

| Strain | TCID_50_ (0.1mL) | EID_50_ (0.1mL) | Genotype | Virulence |
| --- | --- | --- | --- | --- |
| La Sota | 10^7.7^ | 10^9.1^ | II | Avirulent |
| Herts/33 | 10^7.3^ | ND | IV | Virulent |
| F48E8 | 10^7.4^ | 10^9.2^ | IX | Virulent |

ND means not detected.
