# Supplementary material for: Newcastle disease virus promotes spreading infection through vimentin-dependent tight junction injury mediated by MLC/p-MLC activation
Source: PLoS Pathog. 2025 Aug 29;21(8):e1013458. doi: 10.1371/journal.ppat.1013458 (PMC12410888; doi:10.1371/journal.ppat.1013458)
Supplement: S2 Table — (DOCX) [file ppat.1013458.s017.docx]

**S2 Table.** Primer sequences designed for constructing eukaryotic expression plasmids of viral proteins.

| Primer | Sequence (5’–3’) |
| --- | --- |
| NP-F | gagacccaagctgGCTAGC**TCTAGA**ATGTCTTCCGTATTTGATGA |
| NP-R | TCATGGTCTTTGTAGTC**GGATCC**ATACCCCCAGTCGGTGTC |
| P-F | agctgGCTAGC**TCTAGA**ATGGCCACCTTTACAGACG |
| P-R | GGTCTTTGTAGTC**GGATCC**GCCATTCAGCGCAAGGC |
| M-F | ccaagctgGCTAGC**TCTAGA**ATGGACTCATCTAGGACAAT |
| M-R | GGTCTTTGTAGTC**GGATCC**TTTCTTGAAAGGATTGTATTTAG |
| F-F | agacccaagctgGCTAGC**TCTAGA**ATGGGCCCCAAATCTTCTAC |
| F-R | CATGGTCTTTGTAGTC**GGATCC**CATTCTTGTAGTGGCCCTCATCT |
| HN-F | agacccaagctgGCTAGC**TCTAGA**ATGGACCGTGTAGTTAGCC |
| HN-R | TGGTCTTTGTAGTC**GGATCC**AATCCCATCATCCTTGAGAATCT |
| L-F1 | acccaagctgGCTAGC**TCTAGA**ATGGCGAGCTCCGGTCC |
| L-R1 | CCGAGAGGCCTCCGAGTCAGTGCGATCTTAATCACGGTG |
| L-F2 | GACTCGGAGGCCTCTCGGCATCAGGAGGCTGATGCG |
| L-R2 | TGGTCTTTGTAGTC**GGATCC**AGAGTCACAGTTACTGTAATATCCC |
| V-F | CTAGACTCGAGGT**GAATTC**ATGGCCACCTTTACAGACG |
| V-R | CTTTGTAGTC**TCTAGA**CTTACCCTCTGTGATATCGCCT |
| W-F | CTAGACTCGAGGT**GAATTC**ATGGCCACCTTTACAGACG |
| W-R | TCATGGTCTTTGTAGTC**TCTAGA**TCGCCTGCACAAAGT |

Six structural proteins (NP, P, M, F, HN, and L) contain an XbaI insertion site at their 5’ end and a BamHI insertion site at their 3’ end. Two non-structural proteins (V and W) have an EcoRI cleavage site at their 5’ end and an XbaI cleavage site at their 3’ end. Bold text indicates enzyme cleavage sites, while underlined text represents homologous arms.
