# Supplementary material for: Newcastle disease virus promotes spreading infection through vimentin-dependent tight junction injury mediated by MLC/p-MLC activation
Source: PLoS Pathog. 2025 Aug 29;21(8):e1013458. doi: 10.1371/journal.ppat.1013458 (PMC12410888; doi:10.1371/journal.ppat.1013458)
Supplement: S3 Table — (DOCX) [file ppat.1013458.s018.docx]

**S3 Table.** siRNA sequences designed for the specific knockdown of OCLN and ZO-1 gene

| siRNA | Sequence (5’–3’) |
| --- | --- |
| OCLN (A549) | GTGAAGAGTACATGGCTGC |
| ZO-1 (A549) | GCGATCTCATAAACTTCGTAA |
| OCLN (HD11) | GCGCAGAUGUCCAGCGGUUTT |
| ZO-1 (HD11) | CAGCAAAGGUAUACAGAAATT |
