# Supplementary material for: Newcastle disease virus promotes spreading infection through vimentin-dependent tight junction injury mediated by MLC/p-MLC activation
Source: PLoS Pathog. 2025 Aug 29;21(8):e1013458. doi: 10.1371/journal.ppat.1013458 (PMC12410888; doi:10.1371/journal.ppat.1013458)
Supplement: S4 Table — (DOCX) [file ppat.1013458.s019.docx]

**S4 Table.** Primers sequences used in qPCR assay

| Primer | Sequence (5’–3’) |
| --- | --- |
| OCLN-F | CAGGCCTCTTGAAAGTCCACC |
| OCLN-R | AGGCTGGCTGAGAGAGCATT |
| ZO-1-F | GGGACAACAGCATCCTTCCA |
| ZO-1-R | ATCACAGTGTGGTAAGCGCA |
| IL-1β-F | CTCGCCAGTGAAATGATGGCT |
| IL-1β-R | GTCGGAGATTCGTAGCTGGAT |
| IL-6-F | GGAGACTTGCCTGGTGAA |
| IL-6-R | GCATTTGTGGTTGGGTCA |
| IL-18-F | AAACTATTTGTCGCAGGAATAAAG |
| IL-18-R | GCTTGCCAAAGTAATCTGATTCC |
| TNF-α-F | TTCTCCTTCCTGATCGTGG |
| TNF-α-R | AAGATGATCTGACTGCC |
| GAPDH-F | GAAGGTCGGAGTCAACGGATTT |
| GAPDH-R | ATCTTGAGGCTGTTGTCATACTTCT |
