# Supplementary material for: Newcastle disease virus promotes spreading infection through vimentin-dependent tight junction injury mediated by MLC/p-MLC activation
Source: PLoS Pathog. 2025 Aug 29;21(8):e1013458. doi: 10.1371/journal.ppat.1013458 (PMC12410888; doi:10.1371/journal.ppat.1013458)
Supplement: S5 Table — (DOCX) [file ppat.1013458.s020.docx]

**S5 Table.** Virus shedding in oropharyngeal and cloacal swabs of chickens, determined by inoculating SPF chicken embryos

| Days post infection | No. of chickens shedding/total no. of chickens | | | | | |
| --- | --- | --- | --- | --- | --- | --- |
|  | F48E8 | | La Sota | | PBS | |
|  | O^a^ | C^b^ | O | C | O | C |
| 1 | 0/6 | 0/6 | 0/6 | 0/6 | 0/3 | 0/3 |
| 2 | 4/6 | 0/6 | 0/6 | 0/6 | 0/3 | 0/3 |
| 3 | 6/6 | 5/6 | 3/6 | 0/6 | 0/3 | 0/3 |
| 4 | 6/6 | 6/6 | 6/6 | 1/6 | 0/3 | 0/3 |

^a^ oropharyngeal swabs

^b^ cloacal swabs
