# Supplementary material for: Newcastle disease virus promotes spreading infection through vimentin-dependent tight junction injury mediated by MLC/p-MLC activation
Source: PLoS Pathog. 2025 Aug 29;21(8):e1013458. doi: 10.1371/journal.ppat.1013458 (PMC12410888; doi:10.1371/journal.ppat.1013458)
Supplement: Attachment — Submitted filename: Response Letter.docx [file ppat.1013458.s022.docx]

Dear Editor,

Thank you for your decision letter concerning our manuscript (**PPATHOGENS-D-24-02713**) entitled “**Newcastle disease virus promotes spreading infection through vimentin-dependent tight junction injury mediated by MLC/p-MLC activation**”, and your time regarding for our revision. We sincerely appreciate the critical comments from both you and the reviewers. We have carefully considered all feedback and revised the manuscript accordingly. To strengthen our conclusions, we performed essential experiments and incorporated relevant references to support our findings. We have also conducted extensive optimization of statements throughout the text. We believe these modifications enhance the article’s depth and clarity. We hope the revised version now meets the journal’s standards. All modifications are indicated in red text. Below is our point-by-point response to the comments and a detailed list of changes.

Best regards,

Xiaowen Liu

Point-by-point responses to the comments of the reviewer, and a list of changes are:

**Additional Editor Comments:**

Dear Dr. Xiaowen Liu,

Thank you for your patience during the peer review process for your manuscript, “Newcastle disease virus facilitates viral replication and spread through vimentin-dependent tight junction injury”. The manuscript has now been evaluated by reviewers with relevant expertise.

As detailed in their comments below, the reviewers raise significant concerns regarding the conclusiveness of the study. In light of these evaluations, we regret to inform you that we can only consider a revised version of your manuscript, provided that all reviewers’ concerns are thoroughly addressed.

We acknowledge that addressing these points will require substantial additional work, and we understand that this may lead to modifications of your current conclusions. As such, we cannot guarantee acceptance of the revised manuscript.

**Response:** We sincerely thank you and the reviewers for your time and valuable feedback on our manuscript. We have carefully read the editors’ and reviewers’ comments and understand their concerns about the study’s conclusiveness. While we recognize that addressing these points will be a significant undertaking, we are fully committed to revising the manuscript thoroughly to address all concerns raised. We appreciate the opportunity to improve our work and hope that a revised version, incorporating these changes, will meet your journal’s standards.

**Model choice:** There are concerns about the use of A549 epithelial cells and HD11 chicken macrophages as infection models. Please either justify the use of these models or consider repeating key experiments using more physiologically relevant systems, as NDV does not infect humans and macrophages’ TJ are limited.

**Response:** Thanks for your valuable suggestions. We selected A549 (human lung epithelial cells) and HD11 (chicken macrophages) cell lines based on prior research and screening. A549 cells are a proven model for NDV pathogenesis due to their susceptibility for NDV and established use in tight junction research. Studies by Yang et al. (https://doi.org/10.1371/journal.ppat.1012531, *PLoS Pathogens*), Liu et al. (https://doi.org/10.1080/21505594.2022.2112821, *Virulence*), and Li et al. (https://doi.org/10.1016/j.vetmic.2024.110288, *Veterinary Microbiology*) highlight their value for investigating NDV mechanisms. Their tight junction properties, confirmed by Peter et al. (https://doi.org/10.1002/mc.20485, *Molecular Carcinogenesis*), and extensive use in junctional studies [e.g., Iqbal et al. (https://doi.org/10.1093/glycob/cwae093, *Glycobiology*); Cui et al. (https://doi.org/10.1080/15548627.2024.2330043, *Autophagy*); Eguchi et al. (https://doi.org/10.1038/s41598-018-33566-w, *Scientific Reports*); Liu et al. ( https://www.medsci.org/v20p0581.htm, *Int J Med Sci*)]. Therefore, A549 is an ideal cell model for examining NDV’s effects on tight junctions.

For the avian cells, HD11 cells were chosen for their tight junction formation, providing a necessary avian cell model. While epithelial cells remain the primary and most established models for studying tight junctions, this does not preclude the use of non-epithelial cells. In fact, any cell type capable of forming tight junction-like structure—something observed in certain non-epithelial cells alongside abundant tight junction protein expression—can serve as a candidate research model for this purpose (https://doi.org/10.1038/s41467-022-31587-8, *Nature Communications*; https://doi.org/10.1083/jcb.143.2.391, *Journal of Cell Biology*; https://doi.org/10.1073/pnas.0630649100, *PNAS*). In this study, we initially aimed to use avian-derived epithelial cells, such as CTE cells (a type of chicken tracheal epithelial primary cells). However, the challenges associated with culturing primary cells limited us to preliminary investigations using this cell type. Due to the absence of appropriate avian-derived epithelial cell lines, we examined the modified chicken macrophage line HD11, a susceptible target cell line for NDV. Fortunately, we found that HD11 cells stably express tight junction proteins and form tight junction-like structures, making them a viable model.

Furthermore, to ensure the broad applicability of our findings, we extended our investigation to include HeLa cells (human cervical cancer epithelial cells) and CTE cells (chicken tracheal epithelial cells) during the revision process **(as depicted in S2 Fig)**. While our data confirmed that NDV indeed compromised tight junction integrity across all tested cell lines, we ultimately focused our primary analyses on A549 and HD11 cells. This decision was driven by two practical considerations: the inherent instability of tight junctions in HeLa cells and the significant challenges associated with culturing primary CTE cells. We have clarified the selection criteria for A549 and HD11 cells to provide readers with a better understanding of our experimental design **(lines 411—428, 865—889)**.

**Discussion section:** The current discussion largely reiterates the results and does not adequately explore the mechanisms behind vimentin-mediated tight junction damage. It would benefit from a substantial rewrite to focus on interpretation, underlying mechanisms, and broader implications.

**Response:** Thank you for pointing this out. We have comprehensively revised the discussion section, significantly expanding its scope and deepening its analysis. This provides a clearer explanation of the underlying mechanisms and the significance of our study **(lines 852—987)**.

**Mechanistic insight:** The manuscript does not identify which viral proteins or infection processes are responsible for the observed vimentin-mediated TJ injury. Clarifying this point would significantly strengthen your findings.

**Response:** We greatly appreciate your suggestions. Indeed, our previous research overlooked the clear impact of viral proteins on tight junction function. Following your suggestion, we have added experiments to the revised manuscript to assess this impact after viral protein transfection. We primarily used western blotting to monitor changes in tight junction protein levels. Interestingly, our results indicate that NDV’s effect on tight junctions isn’t due to a single protein but rather a synergistic action of multiple viral proteins, each influencing different tight junction components. For example, NP proteins significantly affect OCLN, whereas F and HN proteins impact ZO-1 **(as depicted in Fig 1H, lines 215—229, 442—453)**.

These findings confirm that the underlying mechanism by which NDV regulates tight junctions is complex, involving the coordinated action of various viral proteins. This complexity explains why current research often focuses on the whole virus rather than individual proteins. We plan to further investigate these protein-specific mechanisms in future studies. Additionally, we will explore the interaction between viral protein functions and specific stages of viral infection to clarify how these stages influence tight junction dynamics.

Your suggestion has been very helpful in guiding our future research direction. Thank you again.

**Additional TJ markers:** Inclusion of data on other tight junction proteins would enhance the robustness of your conclusions.

**Response:** Thanks for your valuable suggestions. We selected OCLN and ZO-1 as primary tight junction targets based on prior screening. Briefly, OCLN, ZO-1, and CLDN1 are classic tight junction proteins. Our previous measurements across various cell lines (mammal: A549 and HeLa cells; avian: HD11 and CTE cells) showed that OCLN and ZO-1 are consistently expressed and significantly downregulated by NDV infection. In contrast, CLDN1 expression was unstable, detected only in A549 and CTE cells. Notably, it was absent in HeLa cells (consistent with previous studies by Cunniffe et al., 2012, *British journal of biomedical science,* PMID: 23304789) and, importantly, also in HD11 cells (a novel observation for this cell line). We have included these screening results in the revised manuscript to clarify our rationale **(S3 Fig, lines 419—426)**. Consequently, we focused on the more consistently expressed OCLN and ZO-1. Given that OCLN and ZO-1 are widely established as principal targets for tight junction studies [e.g., Guo et al. (https://doi.org/10.1016/j.vetmic.2023.109954, *Veterinary Microbiology*); Hu et al. (https://doi.org/10.1186/s12967-024-05376-4, *Journal of Translational Medicine*); Wang et al. (https://doi.org/10.1016/j.phymed.2024.156111, *Phytomedicine*)], employing them in our research is a well-founded approach.

**Central claim:** Most critically, the manuscript does not convincingly establish a causal link between vimentin-mediated TJ injury and enhanced NDV replication and transmission. This point must be addressed with additional evidence or revised interpretations.

**Response:** We are grateful for the insightful suggestions you provided. As suggested, we expanded our study to include potential pathway molecules linked to NDV replication and spread via vimentin-mediated tight junction injury, notably the MLC/p-MLC pathway (**Fig 6**). We have also reorganized the manuscript’s structure and logic for greater clarity, resulting in more profound conclusions. The revised research ideas are as follows:

1. **Model Selection & Initial Findings:** We first found that NDV infection may affect the tight junction function of cells (decreasing TEER and increasing cell migration). We subsequently identified suitable cell models (A549 and HD11) and key tight junction proteins (OCLN, ZO-1). Experiments revealed that velogenic NDV strains disrupt tight junction integrity through multiple protein degradation pathways, a process involving the synergistic effects by several viral proteins. In contrast, the lentogenic strain does not exhibit this effect. This indicates a higher risk of tight junction injury by velogenic NDV.
2. **Functional Confirmation:** We then confirmed that tight junction proteins inhibit NDV replication and spread. Conversely, NDV promotes its own replication and spread by downregulating these tight junction proteins.
3. **Structural Mechanism:** Further investigation revealed that vimentin rearrangement exacerbates tight junction injury, thereby facilitating NDV replication and spread.
4. **Core Molecular Mechanism:** Deeper mechanistic studies showed NDV activates the MLC/p-MLC pathway, triggering vimentin rearrangement and TJ disruption to promote its replication and spread. This effect is reversible with the MLC inhibitor Blebbistatin, establishing MLC/p-MLC as a central regulatory hub in this process.
5. **Broad Applicability:** Finally, we confirmed that this vimentin-mediated TJ disruption mechanism applies broadly to both mammalian and avian cells.

We believe that these supplementary experiments and improved manuscript frameworks provide the necessary evidence to clearly establish a causal link between vimentin-mediated tight junction injury and enhanced NDV replication and transmission.

We hope these comments help guide your decision about how to proceed. Should you choose to undertake the necessary revisions, please add the reference PPATHOGENS-D-24-02713 to facilitate the choice of academic editor and the review process.

**Response:** Thanks. We have carefully revised the manuscript based on the editors’ and reviewers’ valuable suggestions, significantly improving its quality and meeting publication standards.

**Journal Requirements:**

1) Please provide an Author Summary. This should appear in your manuscript between the Abstract (if applicable) and the Introduction, and should be 150-200 words long. The aim should be to make your findings accessible to a wide audience that includes both scientists and non-scientists. Sample summaries can be found on our website under Submission Guidelines: <https://journals.plos.org/plospathogens/s/submission-guidelines#loc-parts-of-a-submission>

**Response:** Thanks. As requested, we have included an Author Summary in the revised manuscript.

2) Please upload all main figures as separate Figure files in .tif or .eps format. For more information about how to convert and format your figure files please see our guidelines:

<https://journals.plos.org/plospathogens/s/figures>

**Response:** Thanks. All main figures have been uploaded as separate Figure files in .tif format.

3) Some material included in your submission may be copyrighted. According to PLOSu2019s copyright policy, authors who use figures or other material (e.g., graphics, clipart, maps) from another author or copyright holder must demonstrate or obtain permission to publish this material under the Creative Commons Attribution 4.0 International (CC BY 4.0) License used by PLOS journals. Please closely review the details of PLOSu2019s copyright requirements here: PLOS Licenses and Copyright. If you need to request permissions from a copyright holder, you may use PLOS's Copyright Content Permission form.

Please respond directly to this email and provide any known details concerning your material's license terms and permissions required for reuse, even if you have not yet obtained copyright permissions or are unsure of your material's copyright compatibility. Once you have responded and addressed all other outstanding technical requirements, you may resubmit your manuscript within Editorial Manager.

Potential Copyright Issues:

- Figures 1, 3, and 9. Please confirm whether you drew the images / clip-art within the figure panels by hand. If you did not draw the images, please provide (a) a link to the source of the images or icons and their license / terms of use; or (b) written permission from the copyright holder to publish the images or icons under our CC BY 4.0 license. Alternatively, you may replace the images with open source alternatives. See these open source resources you may use to replace images / clip-art:

- https://commons.wikimedia.org

- <https://openclipart.org/.>

**Response:** Thank you for pointing this out. Figures 1, 3, and 9 from the original manuscript were drawn by me using the BioRender App and are licensed. Due to revisions, they now appear as Figures 1B, 1D, 3A, and 7 in the revised version, with permission from the copyright holder to publish the images under the CC BY 4.0 license added to each figure caption **(Figs 1B, 1D, 3A, and 7, lines 379, 387—388, 571—572, and 1001)**. Furthermore, we provide a PDF copy of the CC BY 4.0 Licence as provided by BioRender as **Supporting Information** via the online submission system.

4) Please ensure that the funders and grant numbers match between the Financial Disclosure field and the Funding Information tab in your submission form. Note that the funders must be provided in the same order in both places as well.

- State the initials, alongside each funding source, of each author to receive each grant. For example: "This work was supported by the National Institutes of Health (####### to AM; ###### to CJ) and the National Science Foundation (###### to AM)."

- State what role the funders took in the study. If the funders had no role in your study, please state: "The funders had no role in study design, data collection and analysis, decision to publish, or preparation of the manuscript.".

If you did not receive any funding for this study, please simply state: u201cThe authors received no specific funding for this work.u201d

**Response:** Thanks. We have carefully checked the information in the Financial Disclosure field and the Funding Information tab **(lines 1013—1020)**.

**Reviewers’ Comments:**

Part I - Summary

Please use this section to discuss strengths/weaknesses of study, novelty/significance, general execution and scholarship.

**Reviewer #1:** In “Newcastle disease virus facilitates viral replication and spread through vimentin dependent tight junction injury,” Lu et al. provide experimental results showing that NDV infection disrupts TJ protein expression of OCLN and ZO-1, which have previously been implicated in intercellular contact. The authors have performed many experiments to show not only that these genes are associated with NDV infection, but that they are important players to increase the pathogen’s virulence within hosts. The experiments include: (1) a transwell assay showing the difference in cell migration between knockdown cells and control, (2) a few experiments (both in-vitro and in-vivo) to implicate OCLN and ZO-1 in cell migration, showing that downregulation decreases cell migration, and viral shedding (3) several experiments, including a microscopy study, to observe TJ structural differences of the vimentin and F-actin fiber between knockdown cells and control. They also provide a lengthy discussion on the pivotal role of vimentin on viral replication. Overall, an impressive amount of interesting work has been done to elucidate the exact mechanisms of NDV infection and virulence within poultry. My recommendation is for a minor revision to further clarify several areas where the text was slightly unclear.

**Response:** We appreciate your summary of the manuscript and encouraging comments. We have tried to consider all suggestions and revised the manuscript based on the suggestions below. We are convinced these modifications will greatly elevate the manuscript’s quality, fulfilling the criteria for publication.

**Reviewer #2:** The authors have studied tight junction injury during NDV infection. They have used lentogenic and velogenic NDV strains in infection models in A549 cells (human epithelial cell line), HD11 cells (chicken macrophage cell line) and chickens. They conclude that NDV replication and spread is facilitated by vimentin-mediated TJ injury.

Virulence of NDV is predominantly determined by the cleavage site of the fusion protein: the F protein of velogenic contains a polybasic cleavage, and can be cleaved by proteases that are present in different tissues. However, other factors will likely contribute to NDV virulence. Surprisingly, the authors do not summarize current knowledge on NDV virulence in either introduction or discusison.

The manuscript contains a large dataset, with a strong focus on analysis of processes that are observed in cells infected with velogenic NDV. However, in my opinion the data do not support a causal relationship between vimentin-mediated TJ injury and NDV replication and spread. Most importantly, the authors do not provide a biologically plausible mechanism by which NDV causes vimentin-mediated TJ injury. Therefore, it is unclear if the observed TJ injury is either cause or effect of velogenic NDV infection. In my opinion, a “precise mechanism governing viral dissemination” (line 26) is not provided. In addition, it is unclear to what extent the observations are specific for NDV.

**Response:** We appreciate the respected Reviewer #2 for the valuable and constructive comments. F protein is indeed a key viral protein affecting NDV’s virulence and pathogenicity, with its cleavage site being the most direct determinant of virulence. We appreciate your insightful comment regarding this aspect. Accordingly, we have briefly added an introduction of factors affecting virus virulence to the revised manuscript **(lines 94—98)**.

Following valuable feedback from editors and reviewers, we conducted additional experiments. These revealed that multiple viral proteins, including NP, F, and HN proteins, can synergistically damage tight junctions—a complex and interesting phenomenon. Recognizing the differential effects of virulent versus avirulent viruses on tight junctions, we have added a discussion of F protein and other viral proteins, focusing on their potential mechanisms **(lines 901—919)**. Future research will specifically examine how these viral proteins affect tight junctions.

We restructured our manuscript for clarity, leading to more significant conclusions. Our revised study demonstrates that virulent NDV strains compromise tight junction integrity through multiple protein degradation pathways and the involvement of various viral proteins, a mechanism distinct from avirulent strains, thereby presenting a heightened risk. We then confirmed that intact tight junctions limit NDV spread, while NDV downregulates these proteins to enhance its own replication and spread. Further investigation revealed that NDV induces tight junction injury by triggering vimentin rearrangement, thereby aiding viral replication and spread. Mechanistically, we found NDV activates the MLC/p-MLC pathway to cause this vimentin rearrangement and tight junction disruption, an effect that can be blocked by an MLC inhibitor, highlighting MLC/p-MLC as a key control point. Finally, we confirmed this vimentin-based mechanism for tight junction disruption applies broadly to both mammalian and avian cells. Thank you again for your helpful suggestions, which made a real difference to the article’s quality.

**Reviewer #3:** This manuscript thorough described the mechanism by which virulent NDV infection induce vimentin-dependent tight junction injury of rearranging vimentin and F-action, which resulted in the degradation of the TJ proteins OCLN and ZO-1. The author conducted in vitro experiments in both human lung epithelial cells and chicken macrophages. In vivo infection of chickens with NDV also showed disruption of TJ of lung epithelial cells. The studies are very well conducted and manuscript is well-written. There are only a few minor comments need to be addressed.

**Response:** We thank the Reviewer #3 for the positive and constructive comments regarding our paper. We have carefully considered all suggestions and revised the manuscript accordingly, as detailed below. I am convinced these modifications will greatly elevate the manuscript’s quality, fulfilling the criteria for publication.

**Part II – Major Issues: Key Experiments Required for Acceptance**

**Reviewer #1:** Major Comments:
L354: What was the rationale for choosing OCLN and ZO-1 protein levels as opposed to other TJ associated proteins?

**Response:** Thanks. Our preliminary screening focused on OCLN, CLDN1, and ZO-1, all recognized classic tight junction proteins. We found that OCLN and ZO-1 were consistently expressed and significantly downregulated by NDV across most cell lines tested, making them the primary candidates for further study. In contrast, CLDN1’s expression was unstable and absent in some cell lines like HeLa and HD11 cells **(S3 Fig, lines 419—426)**. These screening results explain why we focused on OCLN and ZO-1, and they are widely recognized as primary targets in tight junction studies [e.g., Guo et al. (https://doi.org/10.1016/j.vetmic.2023.109954, *Veterinary Microbiology*); Hu et al. (https://doi.org/10.1186/s12967-024-05376-4, *Journal of Translational Medicine*); Wang et al. (https://doi.org/10.1016/j.phymed.2024.156111, *Phytomedicine*) ]. Consequently, it is possible to investigate tight junctions by focusing on OCLN and ZO-1. Thank you once more for your insightful comments.

L372 and L523: One thing that slightly confused me here and in L523 was how exactly the apoptotic pathway is being investigated, when it appears to be an intracellular pathway rather than an intercellular pathway, which was the focus of many of the experiments. It would also be good to identify what these “diverse” pathways may be that the authors write. Furthermore, is there a link between the apoptotic pathway and the cage formation beginning in L447? Clearer links between these results would benefit the clarity of the text.

**Response:** Thank you for your valuable suggestions. Protein degradation generally occurs via three pathways: the caspase hydrolase, autophagy, and proteasome pathways. In this study, we examined their roles in the downregulation of tight junction proteins by NDV. We found that all three pathways contribute, but the caspase hydrolase pathway plays a particularly significant role. Caspases are proteases that catalyze peptide bond hydrolysis, targeting key cellular proteins for degradation (Feng et al., https://doi.org/10.1039/C5SC03363B, *Chemical Science*). This observation aligns with research on influenza infection. Gopal et al. showed that influenza virus triggers epithelial cell apoptosis, degrading TJ proteins (Gopal et al., https://doi.org/10.3389/fimmu.2020.570681, *Frontiers in Immunology*). Although the caspase pathway operates primarily intracellularly, tight junction proteins are cellular components embedded within or spanning the membrane. Our subcellular localization studies further showed that NDV infection induces the redistribution of these tight junction proteins towards the intracellular space. These findings make them accessible targets for caspase-mediated degradation.

We appreciate your insightful suggestions and have accordingly added explanations and discussions concerning the “diversity” pathways in the revised manuscript **(lines 893—901)**. At this moment, exploring the connection between apoptosis and cage formation is somewhat challenging. That is an insightful question, and we plan to investigate the specific mechanisms involved in future research. Thank you again for your constructive comments.

L399: Could you describe in more detail the implications of the time-dependence of the difference in infection? As the viral titer is a cumulative sum, does this mean that there is no discernible difference in the overall concentration of virus proteins (such as NP) between the knockdown cells and control cells eventually? Does this mean that the knockdown cells migrate to more tissues early in the infection, and this is what makes a difference in overall pathogenicity and virulence of infection, even though the overall concentration of virus proteins will eventually be the same?

**Response:** That’s a good question. Indeed, as you pointed out, the impact of tight junction protein knockdown on viral replication is more pronounced during the early stages of infection. This can be attributed to two main factors. Firstly, in the early phase, infected cells following knockdown often migrate rapidly, facilitating viral spread. Secondly, viral loads are typically lower at this stage, making changes in replication levels more readily detectable. In contrast, during the later stages of infection, the virus has already reached peak replication, rendering the system less sensitive to perturbations like protein knockdown. Additionally, cell viability and migration are often weakened at this later stage, which may contribute to the diminished difference observed between the knockdown and control groups.

To address this important observation, we have refined the description in the revised manuscript and included a detailed discussion of these dynamics **(lines 925—929)**. Once again, thank you for your valuable comments.

L512: I am still confused about the significance of observation and F-actin formed a cage around the NP and OCLN aggregates. What makes them “factories”? Is there some sort of input and output of these aggregates?

**Response:** Thank you for the interesting question. Indeed, during various viral infections, the cytoskeleton can form cage-like structures to encapsulate viral components, notably F-actin cages and vimentin cages. For instance, upon SARS-CoV-2 infection, F-actin rearranges to form cage-like structures that help concentrate factors required for progeny virus synthesis, thereby promoting viral replication (https://doi.org/10.1007/s00705-022-05366-1, *Archives of Virology*).

Here, we observed that NDV infection induces F-actin remodeling, leading to the formation of cage-like structures. Viral NP protein and redistributed OCLN protein heavily accumulate within these cages, exhibiting significantly higher fluorescence intensity. In contrast, fluorescence in the outer region of the cage was weaker. When F-actin redistribution is artificially inhibited, F-actin cage formation is prevented. Consequently, NP protein levels decrease, and OCLN redistribution is blocked. These results confirm that F-actin cage formation contributes to NDV replication and OCLN redistribution.

We appreciate your insightful comment. Indeed, numerous studies have established the cytoskeleton cage (such as vimentin cage) as a key site for viral replication. Many RNA and DNA viruses can induce intracellular cage-like structures. For example, during Zika virus infection, vimentin envelops viral envelope proteins, and double-stranded RNA colocalizes with calreticulin and vimentin at perinuclear viral factories (https://doi.org/10.1038/srep44286, *Scientific Reports*). During PRRSV infection, vimentin filaments reorganize into cage-like structures that encase the viral replication complex near the nucleus, thereby establishing a viral factory (https://doi.org/10.1016/j.vetmic.2021.109133, *Veterinary Microbiology*). In this study, we adopt an innovative perspective, viewing F-actin and vimentin cages not as isolated entities, but as collaborating platforms that collectively regulate viral replication and tight junction redistribution. This finding is an interesting discovery in our study, although it is not our primary focus. Our future research will focus on elucidating the specific mechanisms by which F-actin and vimentin cages regulate viral infection, including their impact on the transport of viral and tight junction proteins, viral transfer, and assembly.

To better understand this finding, we have refined the relevant descriptions in the revised manuscript **(lines 604—606, 628—633, and 944—961)**. Thank you once more for your insightful and valuable feedback.

L829: Have other studies been done on other TJ proteins, or other intercellular or intracellular proteins? It would be useful to contextualize these results with other research to understand if they are the sole drivers of NDV infection and pathogenicity, or if there are others.

**Response:** Thank you for your valuable suggestions. We have added analyses of the other tight junction protein, CLDN1. CLDN1 is a classic tight junction protein, frequently used alongside ZO-1 and OCLN as markers in tight junction research. NDV can downregulate these classical tight junction proteins, but the effects on OCLN and ZO-1 are particularly stable and significant, justifying our focus on these two **(lines 419—428, 875—887)**. Our study did not examine other intercellular proteins as they fall outside its scope. We appreciate your constructive suggestion. We will explore the role of other intercellular or intracellular proteins in our future research. In summary, while multiple intercellular factors likely influence NDV infection, we believe tight junctions represent a primary pathway.

**Reviewer #2:** in my opinion this paper does not elucidate a molecular mechanism (as claimed at the end of the discussion), but describes in detail the cytopathic changes in cells that are induced by velogenic NDV infection. There is no evidence for a specific NDV interaction with vimentin or with TJs.

**Response:** We appreciate the reviewer #2’s comment. To enhance the depth and rigor of our research, we have incorporated the suggestions from the editors and reviewers. Consequently, we have added several key experiments, including expanded assays for tight junction proteins, broader cellular analysis, identification of critical viral proteins, and characterization of signaling pathway molecules. Furthermore, we have thoroughly revised the manuscript’s structure to ensure a more logical and coherent presentation of our research approach. The revised research framework is outlined below:

1. **Model Selection & Initial Findings:** NDV infection was found to affect cell tight junction function, decreasing TEER and increasing cell migration. Suitable cell models (A549, HD11) and key tight junction proteins (OCLN, ZO-1) were identified. Experiments showed that velogenic NDV strains disrupt tight junction integrity via multiple protein-degradation pathways, a process involving synergistic viral protein effects. In contrast, the lentogenic NDV strain does not exhibit this effect, indicating a higher risk of tight junction injury with velogenic NDV.
2. **Functional Confirmation & Structural Mechanism:** Tight junction proteins were confirmed to inhibit NDV replication and spread. Conversely, NDV promotes its own replication and spread by downregulating these proteins. Further investigation revealed that vimentin rearrangement exacerbates tight junction injury, facilitating NDV replication and spread.
3. **Core Molecular Mechanism:** Deeper studies showed NDV activates the MLC/p-MLC pathway, triggering vimentin rearrangement and TJ disruption to promote replication and spread. This effect is reversible with the MLC inhibitor Blebbistatin, establishing MLC/p-MLC as a central regulatory hub.
4. **Broad Applicability:** We confirmed that this vimentin-mediated TJ disruption mechanism applies broadly to both mammalian and avian cells.

We used siRNA knockdown and pharmacological inhibition to show that NDV relication and spread are functionally required for vimentin-mediated TJ disruption through MLC/p-MLC activation, although our study did not directly demonstrate physical interactions between viral components and vimentin or tight junctions. These experiments reveal a clear functional link between NDV infection, vimentin reorganization, and tight junction injury. Of note, our findings point to a complex scenario where multiple NDV components contribute to tight junction disruption, rather than a single viral protein being solely responsible. The regulation of different tight junction proteins also varies. This complexity underscores that a full molecular mechanistic understanding will be a significant challenge requiring further work. Our current study, however, provides meaningful insights by focusing on the broad impact of NDV on tight junction integrity and exploring potential pathways of interaction. We view this as an important first step, with detailed dissection of specific molecular interactions reserved for future studies. Once again, thank you for your valuable comments. I am confident that these revisions will significantly enhance the quality of the manuscript, bringing it closer to the standards expected for publication.

**Reviewer #3:** none

**Response:** Thank you for your positive feedback on this research.

**Part III – Minor Issues: Editorial and Data Presentation Modifications**

**Reviewer #1: Minor Comments:**

L71: Should be “However, the infected cells can also be…”?

**Response:** Thanks. We have removed this description after careful consideration, as we found it redundant. We are confident this deletion does not detract from the article’s coherence or completeness; rather, it improves the overall conciseness.

L380: Should be “disrupting” instead of “distrusting”?

**Response:** We appreciate you bringing this to our attention. We have revised the relevant text in that section, and the issue has been resolved.

L456: Not sure why the sentence starts with “Meanwhile,” – maybe “In addition,” would work better? Same with L523

**Response:** Thank you for your insightful suggestion. We concur that “In addition” is the more suitable term here and have made the necessary modifications to the two relevant points (**lines 551 and 617**).

L789: Eliminate “preliminarily”

**Response:** Thanks. We have eliminated it.

**Reviewer #2:** 1. Lines 49-51: the authors suggest that all cells contain TJs. TJs play a crucial role in epithelial barriers (as mentioned in line 51). However, the authors do not discuss the presence, absence or importance of TJs in non-epithelial cells.

**Response:** Thank you for pointing this out. Tight junctions are characteristic of highly polarized epithelial and endothelial cells, serving as key barriers. In contrast, while non-epithelial/endothelial cells are not typically associated with tight junction formation, the tight junction proteins can indeed be highly expressed on such cells and form tight junction-like structure (https://doi.org/10.1038/s41467-022-31587-8, *Nature Communications*; https://doi.org/10.1083/jcb.143.2.391, *Journal of Cell Biology*; https://doi.org/10.1073/pnas.0630649100, *PNAS*). Therefore, epithelial and endothelial cells serve as the primary or most established research models for studying tight junctions. However, this doesn’t mean non-epithelial/endothelial cells are unsuitable. Any cell type capable of forming functional tight junctions can potentially serve as a valuable research candidate model for this purpose.

We have revised these descriptions accordingly and added supporting references (**lines 72—75, 765—766, 882—889**).

2. It has been described that TJ formation in A549 cells is inconsistent (Radiom et al., PMID 33173147). Therefore, it is surprising that the authors have selected this human cell line as their most important model. Velogenic NDV strains do not cause disease in (immunocompetent) humans. Moreover, the second (avian) cell line is a macrophage cell line, a cell type in which TJs play a limited role. The infection models used by the authors should be better explained and justified.

**Response:** That’s a good question. A549 cells are a well-established and widely used model for studying tight junctions, a fact supported by numerous investigations published in internationally renowned journals, including *Autophagy* (https://doi.org/10.1080/15548627.2024.2330043), *Molecular Carcinogenesis* (https://doi.org/10.1002/mc.20485), *Glycobiology* (https://doi.org/10.1093/glycob/cwae093), *Scientific Reports* (https://doi.org/10.1038/s41598-018-33566-w), *International Journal Of Medical Sciences* (https://www.medsci.org/v20p0581.htm). Although NDV primarily infects poultry, it also has the potential to infect humans and cause clinical symptoms (). Significantly, A549 cells have emerged as a valuable model for dissecting NDV pathogenesis, exhibiting robust viral infectivity and replication. Indeed, previous studies using A549 cells have revealed novel mechanisms of NDV infection. For example, Yang et al. used A549 cells to reveal the membrane fusion regulation mechanism of NDV (https://doi.org/10.1371/journal.ppat.1012531, *PLoS Pathogens*), and Liu et al. employed A549 cells to elucidate the replication mechanism of NDV (https://doi.org/10.1080/21505594.2022.2112821, *Virulence*).

While epithelial cells remain the primary and most established models for studying tight junctions, this does not preclude the use of non-epithelial cells. In fact, any cell type capable of forming tight junction-like structure—something observed in certain non-epithelial cells alongside abundant tight junction protein expression—can serve as a candidate research model for this purpose (https://doi.org/10.1038/s41467-022-31587-8, *Nature Communications*; https://doi.org/10.1083/jcb.143.2.391, *Journal of Cell Biology*; https://doi.org/10.1073/pnas.0630649100, *PNAS*). In this study, we initially aimed to use avian-derived epithelial cells, such as CTE cells (a type of chicken tracheal epithelial primary cells). However, the challenges associated with culturing primary cells limited us to preliminary investigations using this cell type. Due to the absence of appropriate avian-derived epithelial cell lines, we examined the modified chicken macrophage line HD11, a susceptible target cell line for NDV. Fortunately, we found that HD11 cells stably express tight junction proteins and form tight junction-like structures, making them a viable model. In addition, to be more rigorous, we described the structures observed in HD11 cells as “tight junction-like structures” rather than “tight junction structures” throughout the text.

Consequently, our study utilized A549 human lung epithelial cells for the systematic investigation of tight junctions, while HD11 cells served as the avian validation counterpart. Thank you for your suggestion again. We have discussed the rationale behind selecting these two cell types to enhance reader understanding of our approach **(lines 759—770, 865—870, 877—889)**.

3. Line 98: reference 27 does not describe virus infections, while reference 28 describes TJ damage in the context of breaching the blood-brain barrier. The references do not support the statement in the sentence.

**Response:** Thank you for identifying these two issues. We apologize for our oversight in mistakenly identifying the parasite in reference 27 as a virus, and for the fact that reference 28 did not precisely match our description. We have carefully revised the citations accordingly and replaced them with appropriate references (https://doi.org/10.3389/fmicb.2023.1192956, *Frontiers in Microbiology*; https://doi.org/10.1016/j.virol.2019.01.031, *Virology*) (**lines 113 and 114**).

4. Lines 159-169: the authors use a transwell system, but do not specify which transwells were used (what material?) and do not specify the pore size. The principle of the assay migration assay is not clear to me. If this is an assay that has been used before, the authors should provide a reference. With the limited description in this paragraph the assay cannot be reproduced, and the results cannot be interpreted.

**Response:** Thank you for your valuable suggestion. The Transwell assay assesses cell migration by placing cells on a microporous membrane, with a chemotactic agent (such as culture media containing high concentrations of serum) below. Migratory cells move through the pores towards the attractant. By quantifying the number of cells that have traversed the membrane, we can evaluate migration capacity. We have optimized our Transwell protocol, including materials and procedures, and relevant references are provided (**lines 177—190**).

5. Lines 382-383: the data do not support a causal relationship as suggested by the paragraph title. In addition, it is unclear if the observations are specific for NDV.

**Response:** Thank you for your valuable suggestion. To better reflect the research findings, we have revised the title (**lines 455 and 456, “**Reduced expression of TJ proteins facilitates both viral replication and spread of NDV*”*) and strengthened the conclusion for greater rigor (**lines 537—540**). These phenomena have also been observed during infection of other viruses, such as SARS-CoV-2 (https://doi.org/10.1073/pnas.2218623120, *PNAS*), PRRSV (https://doi.org/10.1016/j.vetmic.2023.109890, *Veterinary Microbiology*), HAZV (https://doi.org/10.3389/fmicb.2023.1192956, *Frontiers in Microbiology*). These viruses can downregulate the expression of tight junction proteins. This action damages the integrity of intercellular tight junctions, which inherently provide resistance to viral invasion. By exploiting this characteristic, viruses effectively enhance their capacity for infection. We have added relevant discussion on this topic in the Discussion section **(lines 923—932)**.

6. Is the cage formation described by the authors related to inclusion bodies as described by others? As far as I can see the authors do not link this observation to similar observations in the literature.

**Response:** Thanks. Indeed, during various viral infections, the cytoskeleton can form cage-like structures to encapsulate viral components, notably F-actin cages and vimentin cages. For instance, upon SARS-CoV-2 infection, F-actin rearranges to form cage-like structures that help concentrate factors required for progeny virus synthesis, thereby promoting viral replication (https://doi.org/10.1007/s00705-022-05366-1, *Archives of Virology*). In addition, many RNA and DNA viruses can induce intracellular cage-like structures. For example, during Zika virus infection, vimentin envelops viral envelope proteins, and double-stranded RNA colocalizes with calreticulin and vimentin at perinuclear viral factories (https://doi.org/10.1038/srep44286, *Scientific Reports*). During PRRSV infection, vimentin filaments reorganize into cage-like structures that encase the viral replication complex near the nucleus, thereby establishing a viral factory (https://doi.org/10.1016/j.vetmic.2021.109133, *Veterinary Microbiology*). Therefore, the formation of these cage structures often facilitates viral infection and replication.

This study revealed that NDV infection unexpectedly induces both F-actin and vimentin cage formation simultaneously, with these structures colocalizing. Crucially, inhibiting vimentin rearrangement to form cages also causes the F-actin cages to disappear immediately, suggesting their synergistic generation. Furthermore, NDV particles and redistributed tight junction proteins accumulate heavily within these cages. When cage formation is inhibited, NDV replication significantly decreases, and tight junction rearrangement is also suppressed. These findings indicate that cytoskeletal cage structures play a key role in NDV replication and tight junction remodeling during infection.

We appreciate your suggestion. We have thoroughly discussed the potential functions of the cage structure in this study, incorporating the insights from the references **(lines 944—961)**.

7. Lines 584-585: it is impossible to study inflammation in an in vitro model that lacks immune cells.

**Response:** Thank you for your feedback and your concerns regarding the use of cells for studying inflammation. We appreciate this perspective.Our study investigates how tight junction injury influences the inflammatory response to NDV. We selected A549 human lung epithelial cells and HD11 chicken macrophages for this purpose, and we believe this choice is well-justified. Firstly, A549 cells are relevant because they are primary targets of respiratory viruses like NDV. Previous research confirms that NDV infection activates significant inflammatory responses in A549 cells, making them a standard model for studying NDV-induced inflammation (https://doi.org/10.1016/j.virol.2018.09.001, *Virology*). Secondly, HD11 macrophages are essential immune cells crucial for antiviral responses and inflammation. Studying macrophages is fundamental to understanding the immune system’s role in inflammation (https://doi.org/10.1016/j.cyto.2025.156895, *Cytokine*; <https://doi.org/10.1016/j.psj.2024.104683,> *Poultry Science*).

Therefore, we maintain that our chosen models are appropriate and reliable for studying NDV-induced inflammation. The combined data from A549 and HD11 cells provides a robust and comprehensive understanding of the mechanisms involved.

8. Line 617: the background and mechanism of ajoene as vimentin rearrangememnt activator should be explained and cited in the manuscript.

**Response:** Thank you for your valuable suggestion. We have added further explanation regarding the drug and included relevant references **(lines 712—714)**.

9. Lines 660-664: why do the authors use HD11 cells (a chicken macrophage line) and not an avian epithelial cell line? Macrophages do not normally form tight junctions.

**Response:** Thanks. As previously mentioned (question 2), we initially planned to use primary chicken tracheal epithelial cells (CTE) for tight junction studies. However, CTE cells were difficult to culture and unstable, preventing systematic validation. Since stable avian epithelial cells were unavailable, we evaluated the avian macrophage line HD11, which is susceptible to NDV. Although HD11 isn’t an epithelial cell, it formed tight junction-like structures when confluent, consistent with reports on non-epithelial cells [(https://doi.org/10.1038/s41467-022-31587-8, *Nature Communications*; https://doi.org/10.1083/jcb.143.2.391, *Journal of Cell Biology*; https://doi.org/10.1073/pnas.0630649100, *PNAS*)]. Key tight junction proteins were also stably expressed in HD11, and NDV infection successfully downregulated these proteins and disrupted the junctions. These findings show HD11 can model NDV’s effect on tight junctions. Thus, using HD11 as an avian validation model was a reasonable choice given the lack of alternatives, and subsequent experiments confirmed its feasibility. We have reorganized the text to clearly explain the rationale and feasibility for using HD11 as our avian validation model **(lines 759—768, 882—889)**. In addition, to be more rigorous, we described the structures observed in HD11 cells as “tight junction-like structures” rather than “tight junction structures” throughout the text.

10. The discussion is long, but is mostly a reiteration of the results of the study. Most importantly, the authors do not discuss what viral proteins, viral factors infection processes cause the vimentin-mediated TJ damage (see eg lines 838-839).

**Response:** Thank you for your constructive suggestion. We have carefully incorporated your suggestion by adding data on how viral proteins regulate tight junction proteins **(lines 442—453)**. Furthermore, we have provided a more in-depth discussion of the regulatory role of viral proteins on tight junctions and their potential mechanisms **(lines 901—919)**. These additions significantly enrich the research.

11. NDV virons are formed by budding, so it is unlikely that they accumulate within cytoskeletal cages. Do the authors mean RNPs?

**Response:** Thank you very much for your insightful comment. Yes, NDV virions bud from the cell membrane, making cytosolic accumulation of complete virions within F-actin cages less likely. Our focus on the NP protein was strategic for several reasons. Firstly, NP is the most commonly used marker for NDV detection across various research contexts due to its abundant expression. Its levels provide a reliable proxy for overall viral replication kinetics within the cell. Secondly, as you pointed out, NP is a core component of the ribonucleoprotein (RNP) complex, which is indeed encapsulated by the viral nucleocapsid protein within a helical structure. The interaction we observed between NP (and by extension, the RNP complex) and the cytoskeletal cages could potentially influence RNP assembly, trafficking, or stability during the replication cycle. While RNP formation and function are critical for NDV propagation, our current study primarily aimed to establish a broad correlation between viral replication, indicated by NP expression, and its spatial organization relative to the cytoskeleton. We acknowledge that a more detailed investigation into the specific dynamics and regulatory mechanisms governing the interaction between these cytoskeletal cages and the RNP complex is warranted and represents an important direction for future research.

In summary, the NP protein is a good marker for NDV replication and a key RNP component. We’ve updated the manuscript accordingly and explored these mechanisms in the Discussion **(lines 628—633, 9****57—959)**. Future research will focus on exploring how these cytoskeletal cages interact with the RNP complex.

12. Lines 888-889: the model presented in figure 9 is not convincing. The term “through” in line 889 is not supported by the data.

**Response:** Thank you very much for your valuable suggestions. We have deeply appreciated your insightful feedback, which has highlighted the areas needing improvement regarding the mechanism diagram’s accuracy and the alignment of the descriptions with our experimental findings. We have carefully reevaluated the research approach of the entire manuscript and made comprehensive adjustments accordingly. Specifically, based on your and the editor’s comments, we designed and incorporated a series of key experiments. These experiments not only provide new data support but have significantly enhanced the depth and quality of our research. Leveraging these new experimental results, we have redrawn the mechanism diagram, ensuring every detail is rigorously grounded in the empirical data, aiming for both precision and clarity **(Fig 7, lines 989—1001)**. Concurrently, we have meticulously revised the relevant textual descriptions throughout the manuscript to ensure all statements accurately reflect our experimental observations and conclusions, eliminating any previous discrepancies with the actual results. We sincerely hope you will find these revisions satisfactory and kindly request your review of the updated manuscript.

**Reviewer #3:** -Line 71, “the infected cells can be also susceptible to entry into the bloodstream following shedding” this sentence is not clear.

**Response:** Thank you for highlighting this issue. After careful consideration, we fully agree with your assessment that the sentence in question (“the infected cells can be also susceptible to entry into the bloodstream following shedding”) contained ambiguous wording. Consequently, we have decided to remove it. We believe this deletion not only preserves the article’s logical coherence and informational completeness but also significantly enhances its overall conciseness and readability, resulting in more precise expression. We trust these modifications will contribute to a clearer manuscript.

-Statement in line 395 related to Figure 2F, the increase of NDV protein relative to the ACTB is not prominent as there is also increase in ACTB level.

**Response:** Thank you for your valuable suggestion. We have repeated the Western blot analysis to confirm the reliability of the results **(Fig 2F)**.

-line 456, re-phrase significantly as no statistical significance analysis was performed.

**Response:** We appreciate your insightful comment. In response, we have carefully reviewed and modified the description to eliminate any language suggesting statistical significance, as no such analysis was conducted. This ensures the scientific rigor and accuracy of our reporting (**lines 551 and 552**).

-Treatment of mock infected cells with IDPN should be included

**Response:** Thank you for your valuable feedback regarding the suggestion to include an IDPN-treated uninfected cell group. We understand your intention to add this control group to rule out potential effects of IDPN itself. However, IDPN is a well-established drug known to inhibit vimentin rearrangement, and we have confirmed its non-toxicity to cells through concentration screening. Consequently, its individual effects are relatively well-understood and controllable. The core focus of this experiment is to investigate the effects and mechanisms of IDPN treatment specifically on infected viral cells. The inclusion or exclusion of an IDPN-treated uninfected group would not alter our understanding of the key finding regarding IDPN’s action in the context of infection.

Furthermore, we have reviewed relevant literature. For instance, Zheng et al., when studying PRRSV infection, utilized inhibitors like IDPN and only examined the infected groups (Figs 4, 6, <https://doi.org/10.1016/j.vetmic.2021.109133,> *Veterinary Microbiology*), without including an uninfected control. Similarly, Cheng et al. adopted a comparable experimental design when studying CSFV infection (Fig 9, <https://doi.org/10.1128/jvi.01770-22,> *Journal of Virology*). These studies, like ours, prioritize examining the specific effects of drugs on infected cells.

In response to your valuable suggestion, we have indeed supplemented the results with data from the IDPN-Mock group in key experiments, specifically the confocal microscopy analysis of vimentin structure in A549 and HD11 cells. These results indicate that IDPN treatment does not affect cell viability and vimentin structure in normal cells. We believe this addition strengthens the rigor of our research by allowing for a more comprehensive assessment of IDPN’s potential influence **(Fig 4B and S12, lines 625, 626, 776—779)**.

-Line 602, the observation of “cell number within the viral foci in the normal infection group” should be defined as syncytia formation, as cells fused together via the expression of viral protein to form a multinuclear cell. Therefore it’s not so accurate to describe each nucleus as a single cell. Same with line 630 figure 6G, 7J and 8D and 8I.

**Response:** Thank you for your valuable comments. We understand your interpretation of our observed phenomenon as “cell fusion”. However, we wish to clarify that the “lesions” we quantify post-infection are distinct from simply observing cell fusion. While cell fusion (syncytia formation) can be one component, our ‘lesions’ represent broader, visible areas of cellular disruption on the monolayer resulting from viral infection. These can include cell death, detachment, and morphological changes beyond just fusion. Counting and measuring these lesions allows us to effectively quantify the overall spread range and efficiency of the virus within the cell population. This is a well-established method in virology for assessing viral dissemination, especially for viruses causing significant cytopathic effects. Our goal is to evaluate the overall spread dynamics of NDV, not just isolate cell fusion events.

Furthermore, this approach aligns with published research. For instance, studies by Yumine et al. and Ohta et al. have similarly used lesion formation assays to evaluate viral spread for different viruses (https://doi.org/10.1016/j.virol.2019.01.031, *Virology*; https://doi.org/10.3389/fmicb.2023.1192956, *Frontiers in Microbiology*). These examples validate our methodology.

Therefore, we believe our experimental design is appropriate for assessing viral spread. We have elaborated on the results in this section, providing clear definitions and supporting references to enhance clarity **(lines 515—518)**.

-Figure 6B, it doesn’t seems to change the distribution of F-actin.

**Response:** Thank you for highlighting this issue. F-actin distribution was re-assessed, and representative images are presented. **Fig 5B** shows that ajoene treatment induced a significant redistribution of F-actin.

-Please discuss more of the mechanism of rearrangement of vimentin by viral infection. Such as if/which viral protein, not limited to NDV, interact with vimentin and why action cage can enhance viral replication.

**Response:** Thank you for your constructive suggestion. To address this, we have expanded our investigation to include an examination of the regulatory effects of viral proteins on tight junctions **(lines 442—453)**. This broader perspective helps contextualize viral protein interactions with tight junctions. Furthermore, we have significantly elaborated in the Discussion section. We now provide a more detailed discussion on the potential mechanisms through which viral proteins might influence both tight junction integrity and vimentin reorganization **(lines 901—919)**. Additionally, we have thoroughly discussed the potential mechanisms by which vimentin and F-actin cages might promote viral replication, integrating insights from existing literature to support our interpretation **(lines 945—961)**. We trust that the incorporated supplements and modifications contribute positively to the article’s quality and the research’s depth.

-Please discuss why avirulent NDV doesn’t cause vimentin-dependent TJ injury as much as the virulent NDV does.

**Response:** Thank you for your insightful question. Our findings show that virulent NDV induces vimentin-mediated tight junction damage, whereas the avirulent NDV strain does not. We attribute this difference to the distinct cellular responses elicited by each strain. Virulent NDV typically exhibits higher replication rates and greater cytotoxicity, generating stronger stress signals that likely trigger cytoskeletal rearrangements, including vimentin reorganization, and interfere with tight junction stability. Avirulent NDV, with its milder replication and cellular impact, may not produce sufficient stress to activate this specific vimentin-dependent tight junction disruption pathway. Furthermore, differences in virulence-associated viral proteins (e.g., the F protein cleavage site) could contribute to this effect.

Given the primary focus of our study on virulent NDV due to its severe economic impact, the avirulent strain served as a control, where this differential effect was observed. We have expanded upon these potential mechanisms in the discussion section, as suggested, to enhance the manuscript’s depth **(lines 910—919)**.
